# QSP model for AAV-mediated antibody delivery in rat brain

**DOI:** 10.1007/s10928-026-10051-w

**Published:** 2026-07-18

**Authors:** Shufang Liu, Ekram Ahmed Chowdhury, Shengjia Wu, Guy Meno-Tetang, Dhaval K. Shah

**Affiliations:** 1 Department of Pharmaceutical Sciences, School of Pharmacy and Pharmaceutical Sciences, The State University of New York at Buffalo, Buffalo, NY 14214-8033 USA; 2 Neuroscience, BioPharmaceuticals R&D, AstraZeneca, Cambridge, UK

**Keywords:** Adeno-associated virus (AAV), Brain local injections, Physiologically based pharmacokinetic (PBPK) modeling, Quantitative systems pharmacology (QSP)

## Abstract

Adeno-associated virus (AAV) mediated monoclonal antibody (mAb) expression in the brain represents an exciting one-and-done therapeutic option for many central nervous system (CNS) disorders that are hard to treat following systemic mAb administration. To facilitate the development of such AAV therapies, here we have developed an AAV-transgene-target quantitative systems pharmacology (QSP) model that can simultaneously characterize the disposition of AAV, expressed antibody, therapeutic target, and the mAb-target complex in plasma, tissues, and the brain. The model was built by integrating previously published platform physiologically based pharmacokinetic (PBPK) models for AAV, protein therapeutics, and brain, with special emphasis on transport pathways and transduction mechanisms within the brain. The developed QSP model was able to capture the pharmacokinetics of AAV and the expressed mAb in the systemic circulation and brain tissues following AAV administration via diverse routes in the rats. The model suggested that intra-cisternal magna injection of medium AAV dose or intrastriatal injection of low AAV dose would provide better therapeutic options compared to intravenous administration of high AAV dose. Locally administered AAV was predicted to lead to sufficient antibody exposure and substantial target engagement in cerebrospinal fluid (CSF) and interstitial fluid (ISF), while minimizing systemic AAV and antibody exposure, leading to maximal efficacy and minimal toxicity. Overall, the present work not only supports cross-species and cross-serotypes translational potential of previously published AAV PBPK model, but also highlights application of QSP modeling to guide the development of AAV therapies for neurological disorders.

**Supplementary Information:**

The online version contains supplementary material available at https://doi.org/10.1007/s10928-026-10051-w.

## Introduction

Monoclonal antibodies (mAbs) developed against many protein targets (e.g., amyloid-β, tau etc.) have emerged as viable therapeutic options for central nervous system (CNS) disorders (e.g., Alzheimer’s disease). Similarly, a proprietary target (i.e., TargetX) has emerged as a viable therapeutic target for developing disease-modifying agents for Parkinson’s disease, which is the most prevalent neurodegenerative motor disorder among the elderly. Quite a few mAbs targeting different conformers of TargetX have shown mitigation of pathology in preclinical models but have shown suboptimal results in clinical trials. Successful application of these mAbs requires efficient delivery into the brain. However, the limited permeability of blood-brain-barrier (BBB) and blood-cerebrospinal fluid barrier (BCSFB) has restricted brain interstitial fluid (ISF) and cerebrospinal fluid (CSF) exposures of mAbs to only about 0.2%-0.3% of plasma exposure [1–4]. Therefore, to achieve sufficient and sustainable target engagement in the brain, there is an emerging interest in utilizing adeno-associated viruses (AAVs) to deliver mAbs expressing genes via local brain injection to bypass the BBB and BCSFB [5, 6].

We have investigated AAV vector and expressed anti-TargetX antibody biodistribution in rat brain, following systemic and local brain injections of AAV at various dose levels, details of which are presented in the companion manuscript. The transgene product, called “anti-TargetX mAb”, is a high affinity antibody that can sequester both monomeric and aggregated forms of TargetX in extracellular space and attenuate spreading of TargetX pathology in rats, which is now being developed as a treatment for Parkinson’s disease. Given the difficulty of projecting first-in-human dose of AAVs at present [7], it is crucial to establish a quantitative systems pharmacology (QSP) model framework to identify an optimal AAV regimen (the serotype, the route of administration, and the dose level) to deliver anti-TargetX mAb into patients.

Our group has recently published a series of papers describing two mechanistic models that set the foundation for building such a framework. One is an AAV physiologically based pharmacokinetic (PBPK) model that characterizes whole-body disposition of both the vector and the transgene, following systemic administration of AAV8 and AAV9 vectors delivering a non-targeted mAb in mice [8]. The other is a brain PBPK model that can capture brain subregion disposition of a non-targeted mAb after local administration in different brain regions of rats [9]. In the present study, we further evolved the AAV-mAb PBPK model into a comprehensive QSP model, by harnessing the detailed compartmentalization and transport pathways of the brain PBPK model. The QSP model has been used to characterize blood and organ disposition of both the AAV9 vector and anti-TargetX mAb upon systemic and local injections in rat brain, and to predict target (i.e., TargetX) engagement in different compartments. The generalizability of the platform AAV-mAb PBPK model is also evaluated by calibration of the model using biodistribution data generated with another AAV serotype (i.e., AAV5). The QSP model presented here provides a quantitative platform for development and clinical translation of AAV mediated antibody delivery for the treatment of CNS disorders.

## Methods

### Dataset description

The experimental design and methodology for the AAV and mAb biodistribution study has been detailed in the companion manuscript. Briefly, the study involves 7 groups of treatments in rats: (i) intravenous (IV) administration of 5E12 Vg AAV9 encoding anti-TargetX mAb (AAV9-anti-TargetX mAb), (ii) intra-cisterna magna (ICM) administration of 5E12 Vg AAV9-anti-TargetX mAb, (iii) ICM administration of 1E12 Vg AAV9-anti-TargetX mAb, (iv) ICM administration of 5E11 Vg AAV9-anti-TargetX mAb, (v) intrastriatal (IST) administration of 2.5E11 Vg AAV9-anti-TargetX mAb, (vi) ICM administration of 1E12 Vg AAV9 encoding a non-targeted control antibody (AAV9-control mAb), and (vii) ICM administration of 5E12 Vg AAV5-anti-TargetX mAb. AAVs in blood, brain, lung, liver, and heart were quantified via qPCR, and antibodies in CSF, brain ISF, brain homogenate, lung, liver, and heart were measured via ELISA.

Pharmacokinetics (PK) and target engagement properties of anti-TargetX mAb in rats were obtained from other in-house studies, including: (i) plasma PK of anti-TargetX mAb after 3, 10, 30, and 100 mg/kg IV administration, (ii) CSF PK of anti-TargetX mAb after 3, 10, 30, and 100 mg/kg IV administration, (iii) ISF PK following IV dosing of 30 mg/kg anti-TargetX mAb, and (iv) free TargetX concentrations in CSF upon 30 mg/kg IV dosing of anti-TargetX mAb.

### QSP model structure

**Fig. 1:**
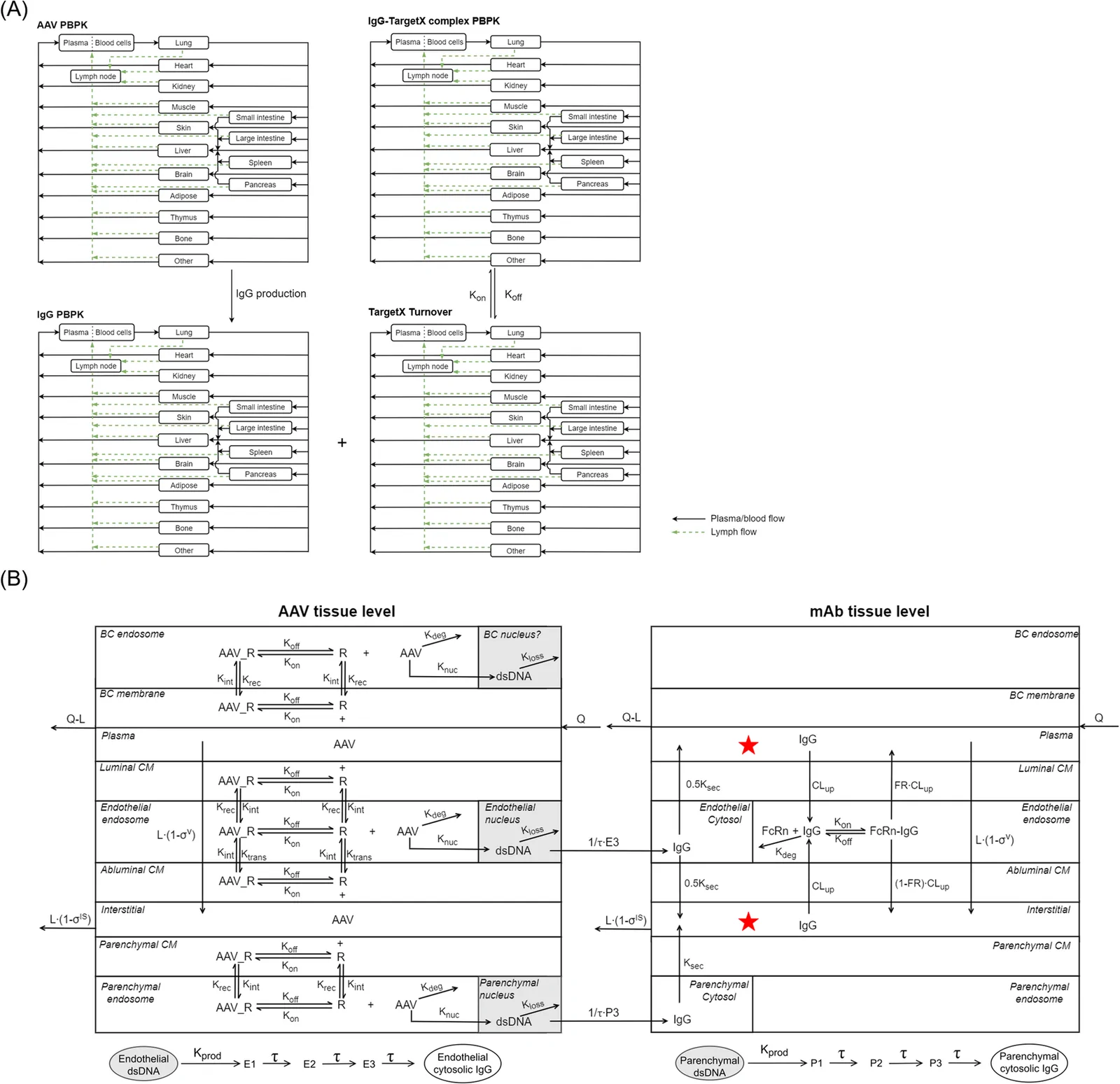
AAV-mAb-TargetX PBPK model structure.** (A) **Whole-body level model structure. The model is comprised of 4 individual PBPK models that describe PK of AAV, transgene-expressed anti-TargetX mAb, endogenously produced TargetX, and formed mAb-target complex, respectively. The AAV model is linked to the antibody PBPK model via transgene expression processes, whereas the antibody PBPK model, the target PBPK model, and the complex PBPK model are connected via kinetic target binding. **(B) **Tissue-level model structure (except for brain). The AAV model incorporates AAV convective transport, receptor-mediated internalization, recycling, and transcytosis, intracellular degradation, nuclear transport, double-stranded DNA formation, and DNA loss in cell nucleus. Then dsDNA in endothelial and parenchymal cells drives anti-TargetX mAb expression in cytosols via a series of transit compartments. Anti-TargetX mAb is subsequently secreted outside cells and follow typical antibody PK characteristics, including convection, nonspecific pinocytosis, FcRn-mediated salvage, and intracellular degradation. The TargetX model and the mAb-target complex model adopt the same model structure as the antibody model, with different PK parameter values. Red stars in the plot represent binding between anti-TargetX mAb and TargetX, which are assumed to occur in liquid compartments including plasma and interstitial spaces

The present AAV-transgene-target model is an enhanced version of the previous AAV-mAb PBPK model coupled with the latest brain submodel [8, 9], with additional features that reflect turnover properties of the target and local transduction of AAVs in the brain. Figure 1A shows the overall model structure composed of 4 PBPK models that characterize whole-body PK of AAV, transgene expressed anti-TargetX mAb, endogenously produced TargetX, and the bound mAb-TargetX complex, respectively. The latter 3 PBPK models adopt the same tissue-level model structure with different PK parameter values associated with neonatal Fc receptor (FcRn) binding and size-dependent elimination mechanisms. These PBPK models are connected to each other via transgene expression or antigen-antibody binding processes.

Figure 1B shows the tissue-level model for 14 tissues except for the brain. Each tissue is divided into 9 compartments: blood cells, blood cell membrane, plasma, endothelial luminal cell membrane, endothelial cells, endothelial abluminal cell membrane, interstitial space, parenchymal cell membrane, and parenchymal cells. Cells can be further segmented into cytoplasmic (or endosomal) space and nucleus for AAV distribution, or into cytoplasmic space and endosomal space for mAb distribution. Vascular AAVs can enter interstitial space via convective transport ($\:\left(1-{\sigma\:}_{org}^{V,AAV}\right)\bullet\:{J}_{org}$). Vascular or interstitial AAVs bind to primary receptors and co-receptors present on blood cells ($\:{R}_{org}^{BC,CM}$), endothelial cells ($\:{R}_{org}^{E,CML}$ and $\:{R}_{org}^{E,CMAL}$), or parenchymal cells ($\:{R}_{org}^{P,CM}$) with a composite binding affinity ($\:{K}_{on}^{AAV}$ and $\:{K}_{off}^{AAV}$), followed by endocytosis ($\:{K}_{int}$) into endosomes. Once inside cells, AAV-receptor complex can either dissociate, recycle ($\:{K}_{rec}$), or, specifically for endothelial cells, transcytose ($\:{K}_{trans}$). Free intracellular AAVs escape from endosomes into cytoplasm and get imported into nucleus, where linear, single-stranded DNA is released from capsids and converted into double-stranded DNA (dsDNA, $\:{K}_{nuc}^{AAV}$). Meanwhile, AAVs are subject to endo-lysosomal protease-mediated degradation and cytoplasmic proteasome-mediated degradation ($\:{K}_{deg}^{AAV}$). In the nucleus, dsDNA can be lost over time ($\:{K}_{loss}^{org}$), due to mechanisms such as cell turnover and DNA degradation. Nuclear DNA in endothelial and parenchymal cells then drive transgene expression ($\:{K}_{prod}^{org}$) via transit compartments ($\:{\tau\:}_{org}$). The produced mAb is folded and then secreted ($\:{K}_{sec}^{org}$) from parenchymal cells into interstitial space, and from endothelial cells into both interstitial and vascular spaces.

Secreted mAb then follows its own PK characteristics. Briefly, vascular antibodies enter interstitial space via lymphatic flow ($\:\left(1-{\sigma\:}_{org}^{V,mAb}\right)\bullet\:{J}_{org}$). Both vascular and interstitial antibodies are nonspecifically pinocytosed into endothelial cells ($\:{CL}_{up}^{org}$), where antibodies can bind to FcRn in acidic endosomes ($\:{K}_{on}^{FcRn,mAb}$ and $\:{K}_{off}^{FcRn,mAb}$), followed by recycling to vascular ($\:{CL}_{up}^{org}\bullet\:FR$) and interstitial space ($\:{CL}_{up}^{org}\bullet\:\left(1-FR\right)$). Interstitial antibodies then leave tissues via lymphatic flow ($\:\left(1-{\sigma\:}_{org}^{I}\right)\bullet\:{J}_{org}$). The mAb-TargetX complex shares the same PK parameters as the mAb in the abovementioned processes. Due to the small size of TargetX, its reflection coefficient that represents tissue resistance to protein convection is smaller ($\:{\sigma\:}_{org}^{V,TargetX}$). It is also assumed that TargetX can diffuse into interstitial space in addition to the convective transport ($\:{Diff}_{TargetX}$), and renal filtration ($\:{CL}_{R}^{TargetX}$) represents an additional pathway of its elimination.

**Fig. 2:**
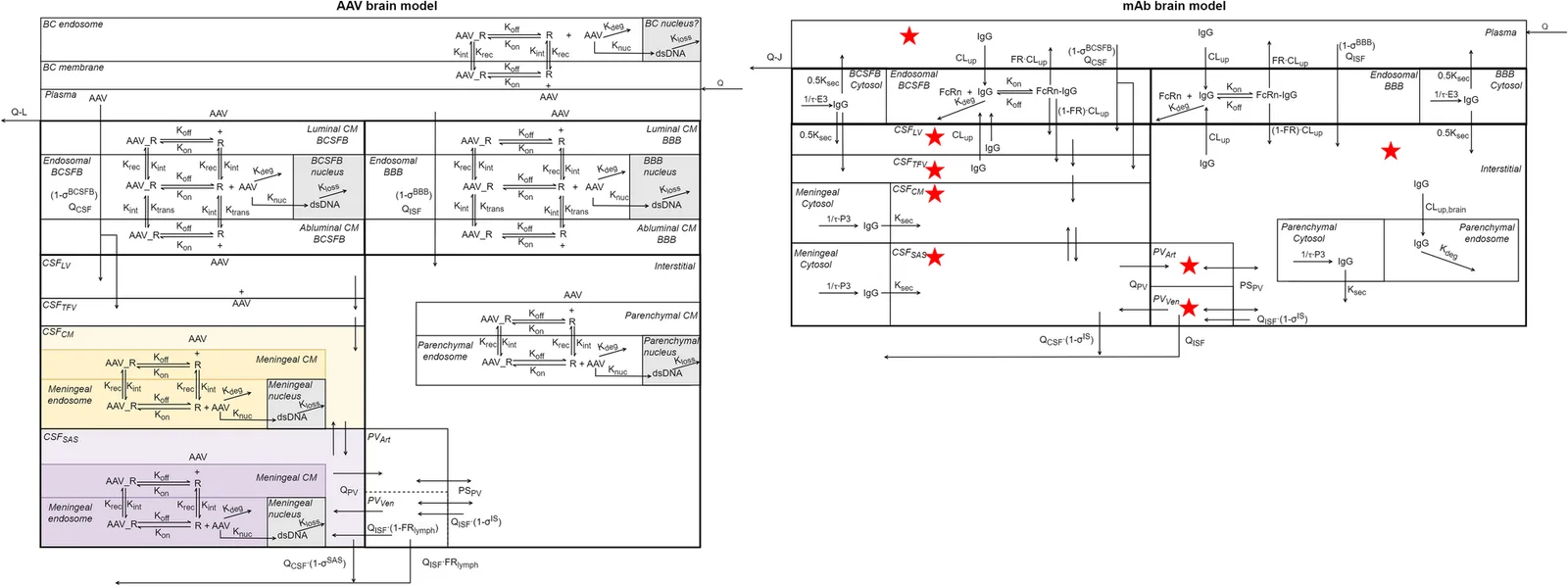
Schematic of the brain PBPK model. In addition to typical compartments and kinetics included in typical organs (described in Fig. 1B), brain has an additional cerebrospinal fluid (CSF) circulation system, and blood-CSF barrier (BCSFB) and blood-brain barrier (BBB) as endothelial cells. CSF is secreted into lateral ventricles (LV) and third-fourth ventricles (TFV) based on their volume ratio. The resultant CSF can be transported from TFV to cisternal magna (CM). There is also an oscillatory CSF flow between CM and subarachnoid space (SAS). Substances in SAS enter peri-arterial space with the perivascular flow, and transport between perivascular space and interstitial fluid (ISF) occurs via diffusion. An ISF flow leaves interstitial space and is directed to the lymph or back to SAS. Expressed mAb can be secreted from parenchymal cells to interstitial space, from meningeal cell to CM and SAS, from BBB cells to interstitial space and plasma space, or from BCSFB to plasma space, LV space, and TFV space. Red stars in the plot represent binding between anti-TargetX mAb and TargetX that occurs in vascular, interstitial, and perivascular spaces, as well as all CSF compartments

The brain model with more detailed compartmentalization is shown in Fig. 2. AAVs in brain plasma space can travel to the lateral ventricle (LV) and the third-fourth ventricle (TFV) through the CSF flow ($\:\left(1-{\sigma\:}_{BCSFB}^{V,AAV}\right)\bullet\:{Q}_{CSF}$) or receptor-mediated transcytosis across BCSFB ($\:{K}_{trans}$). Alternatively, vascular AAVs enter ISF via the ISF flow ($\:\left(1-{\sigma\:}_{BBB}^{V,AAV}\right)\bullet\:{Q}_{ISF}$) or transcytosis across BBB. Once in LV or TFV, besides binding to the abluminal AAV receptors present on BCSFB ($\:{R}_{BCSFB}^{E,CMAL}$), AAVs distribute throughout the CSF space with the CSF flow to cisterna magna (CM) and then the subarachnoid space (SAS). In SAS, AAVs can transit back to CM via an oscillatory CSF flow ($\:{Q}_{CSF}^{OSC}$), recycle to systemic circulation via lymphatic flow ($\:\left(1-{\sigma\:}_{SAS}^{I}\right)\bullet\:{Q}_{CSF}$), or enter peri-arterial space via the perivascular flow ($\:{Q}_{PV}$). Importantly, in CM and SAS, AAVs are assumed to transduce surrounding cells (i.e., meningeal cells). AAVs in perivascular (both peri-arterial and peri-venous) space can be exchanged with those in ISF space via diffusion ($\:{PS}_{PV}^{AAV}$), yet no transport from peri-arterial to peri-venous space occurs. AAVs in ISF can also bind to abluminal AAV receptors on BBB ($\:{R}_{BBB}^{E,CMAL}$), bind to receptors on parenchymal cell membrane ($\:{R}_{Brain}^{P,CM}$), or exit via the ISF flow ($\:\left(1-{\sigma\:}_{Brain}^{I}\right)\bullet\:{Q}_{ISF}$) through the peri-venous space directed to SAS ($\:1-{FR}_{lymph}^{AAV}$) or to the lymph ($\:{FR}_{lymph}^{AAV}$). Processes including receptor-mediated endocytosis and recycling, intracellular processing of AAV, and transgene expression and secretion in BBB, BCSFB, meningeal cells, and brain parenchymal cells were modeled in a similar manner to other tissues. Transport pathways of the produced antibody, target, and the antibody-target complex are the same as those for AAVs, including convection alongside CSF flow, ISF flow, and perivascular flow, as well as exchange between perivascular space and ISF. FcRn-mediated salvage of the antibody occurs in BCSFB and BBB endosomes. Additionally, proteins (anti-TargetX mAb, TargetX, and the bound complex) can be taken up by brain parenchymal cells via pinocytosis ($\:{CL}_{up,brain,cell}$) and get degraded.

It is assumed that TargetX synthesis occurs in central plasma ($\:{Ksyn}_{Plasma}^{TargetX}$), LV ($\:{Ksyn}_{LV}^{TargetX}$), and ISF ($\:{Ksyn}_{ISF}^{TargetX}$). Binding between anti-TargetX mAb and TargetX was assumed to occur in all liquid compartments including central plasma, lymph, organ vascular space, organ interstitial space, brain CSF (LV, TFV, CM, and SAS), and perivascular space.

### Model parameters

Table 1 lists definitions and values of all variables/parameters associated with AAV9 biodistribution, transgene expression and secretion, and the mAb and the target biodistribution.

**Table 1:** Glossary of parameters and variables of the AAV9-anti-TargetX QSP model

Variable	Unit	Definition	Value	Source
AAV biodistribution				
$\:{C}_{Plasma}^{AAV}$,$\:{C}_{Lymph}^{AAV}$	copy/L	AAV concentrations in the central plasma and lymph nodes	Dynamic	-
$\:{V}_{org}^{CV,E}$,$\:{V}_{org}^{CV,P}$	L	Cell volumes of endothelial cells and parenchymal cells in an organ	$$\:\begin{array}{c}V_{org}^{CV,E}=0.07\times\:V_{Org}^C\\V_{org}^{CV,P}=0.93\times\:V_{Org}^C\end{array}$$	[10]
$$\begin{array}{c}C_{org}^{V,AAV},\:C_{org}^{I,AAV},\\C_{org}^{E,E,AAV},\:C_{org}^{BC,E,AAV},\:C_{org}^{P,E,AAV},\\\:C_{org}^{E,N,AAV},\:C_{org}^{P,N,AAV},\:C_{org}^{BC,N,AAV}\\\end{array}\:\:$$	copy/L	AAV concentrations in the vascular, interstitial, endothelial intracellular, blood cell intracellular, parenchymal intracellular, endothelial nuclear, parenchymal nuclear, and blood cell nuclear space for an organ	Dynamic	-
$\:{C}_{BBB}^{E,E,AAV}$,$\:{C}_{BCSFB}^{E,E,AAV}$	copy/L	AAV concentrations in intracellular space of BBB and BCSFB	Dynamic	-
$\:{C}_{BBB}^{E,N,AAV}$,$\:{C}_{BCSFB}^{E,N,AAV}$	copy/L	AAV concentrations in nuclear space of BBB and BCSFB	Dynamic	-
$\:{C}_{Brain}^{SAS,\:AAV}$,$\:{C}_{Brain}^{LV,AAV}$,$\:{C}_{Brain}^{TFV,\:AAV}$,$\:{C}_{Brain}^{CM,AAV}$	copy/L	AAV concentrations in SAS, LV, TFV, and CM compartments	Dynamic	-
$\:{C}_{CM}^{MEN,E,AAV}$,$\:{C}_{SAS}^{MEN,E,AAV}$	copy/L	AAV concentrations in meningeal intracellular space in CM and SAS	Dynamic	-
$\:{C}_{CM}^{MEN,N,AAV}$,$\:{C}_{SAS}^{MEN,N,AAV}$	copy/L	AAV concentrations in meningeal nuclear space in CM and SAS	Dynamic	-
$\:{C}_{Brain}^{PV,Art,AAV}$,$\:{C}_{Brain}^{PV,Ven,AAV}$	copy/L	AAV concentrations in perivascular arterial and venous space	Dynamic	-
$\:{R}_{org}^{E,CML}$,$\:{R}_{org}^{E,CMAL}$,$\:{R}_{org}^{P,CM}$,$\:{R}_{org}^{BC,CM}$	copy/L	Free AAV receptor concentrations on the endothelial luminal cell membrane, endothelial abluminal cell membrane, parenchymal cell membrane, and blood cell membrane of an organ	Dynamic	-
$\:{R}_{CM}^{MEN,CM}$,$\:{R}_{SAS}^{MEN,CM}$	copy/L	Free AAV receptor concentrations on meningeal cell membrane in CM and SAS	Dynamic	-
$\:{R}_{BBB}^{E,CML}$,$\:{R}_{BCSFB}^{E,CML}$	copy/L	Free AAV receptor concentrations on endothelial luminal cell membranes of BBB and BCSFB	Dynamic	-
$\:{R}_{BBB}^{E,CMAL}$,$\:{R}_{BCSFB}^{E,CMAL}$	copy/L	Free AAV receptor concentrations on endothelial abluminal cell membranes of BBB and BCSFB	Dynamic	-
$\:{R}_{org}^{E,\:E}$,$\:{R}_{org}^{P,\:E}$,$\:{R}_{org}^{BC,E}$	copy/L	Free AAV receptor concentrations in the endothelial intracellular, parenchymal intracellular, and blood cell intracellular space of an organ	Dynamic	-
$\:{RC}_{org}^{E,CML}$,$\:{RC}_{org}^{E,CMAL}$,$\:{RC}_{org}^{P,\:CM}$,$\:{RC}_{org}^{BC,CM}$	copy/L	AAV-receptor complex concentrations on the endothelial luminal cell membrane, endothelial abluminal cell membrane, parenchymal cell membrane, and blood cell membrane of an organ	Dynamic	-
$\:{RC}_{CM}^{MEN,CM}$,$\:{RC}_{SAS}^{MEN,CM}$	copy/L	AAV-receptor complex concentrations on meningeal cell membrane in CM and SAS	Dynamic	-
$\:{RC}_{BBB}^{E,CML}$,$\:{RC}_{BCSFB}^{E,CML}$	copy/L	AAV-receptor complex concentrations on endothelial luminal cell membranes of BBB and BCSFB	Dynamic	-
$\:{RC}_{BBB}^{E,CMAL}$,$\:{RC}_{BCSFB}^{E,CMAL}$	copy/L	AAV-receptor complex concentrations on endothelial abluminal cell membranes of BBB and BCSFB	Dynamic	-
$\:{RC}_{org}^{E,\:E}$,$\:{RC}_{org}^{P,\:E}$,$\:{RC}_{org}^{BC,E}$	copy/L	AAV-receptor complex concentrations in the endothelial intracellular, parenchymal intracellular, blood cell intracellular space of an organ	Dynamic	-
$\:{R}_{CM}^{MEN,E}$,$\:{R}_{SAS}^{MEN,E}$	copy/L	Free AAV receptor concentrations in the meningeal intracellular space in CM and SAS	Dynamic	-
$\:{RC}_{CM}^{MEN,E}$,$\:{RC}_{SAS}^{MEN,E}$	copy/L	AAV-receptor complex concentrations in the meningeal intracellular space in CM and SAS	Dynamic	-
$\:{FR}_{lymph}^{AAV}$	-	Fraction of AAVs in perivenous space that flow to the lymph (instead of to SAS)	0	***Fixed***
$\:{R}_{tot}^{org}$	copy/L	Total AAV receptor(s) concentrations in endothelial and parenchymal cells for an organ (initial condition of$\:{R}_{org}^{E,CML}$,$\:{R}_{org}^{E,CMAL}$,$\:{R}_{org}^{P,CM}$)	Liver: 1.6E15 Adipose: 3.3E14 Heart: 3.3E14 Lung: 3.3E14 Spleen: 3.3E14 Muscle: 3.3E14 Kidney: 6.6E13 Skin: 6.6E13 Pancreas: 6.6E13 Brain: 6.6E13 Bone: 6.6E13 ***Estimated***: *Brain meningeal: 3.5E15 *Blood cells: 7.12E11	[8]
$\:{K}_{D}^{AAV}$	copy/L	Equilibrium dissociation constant of AAV binding to its cell-surface receptor(s)	7.2E13 (Equivalent to 120 pM)	[8]
$\:{K}_{on}^{AAV}$	L/copy/h	Association rate of AAV binding to its receptor(s)	$\:\frac{{K}_{off}^{AAV}}{{K}_{D}^{AAV}}$	***Calculated***
$\:{K}_{off}^{AAV}$	1/h	Dissociation rate of AAV binding to its receptor(s)	0.095	[8]
$\:{K}_{deg}^{AAV}$	1/h	Degradation rate of AAV capsids and genome in endosomes and cytosol	1.007	[8]
K_int_	1/h	Internalization rate constant of AAV receptors and the AAV-receptor complex	1.0	[8]
K_rec_, K_trans_,	1/h	Recycling rate constant and transcytosis rate constant of intracellular AAV receptors and the AAV-receptor complex	1.0	[8]
$\:{K}_{nuc}^{AAV}$	1/h	A hybrid parameter that describes the net rate of dsDNA formation from capsid-protected vector genome in endosome, encompassing endosomal escape, nuclear translocation, uncoating, and second-strand DNA synthesis	0.1	[8]
$\:{K}_{loss}^{org}$	1/h	Disappearance rate constant of nuclear transgene DNA	Lung: 4.95E-3 Spleen: 6.9E-3 Muscle: 3.35E-3 Adipose: 4.6E-3 Brain: 0 Liver: 2.7E-3 Kidney: 2.8E-3 Pancreas: 2.8E-3 Skin: 3.84E-3 Bone: 3.97E-3 Heart: 9.1E-4 Blood cells: 8.7E-4 Other organs: 0 All organs: 0 after 21 days	[8]
**Transgene expression**				
$\:{K}_{prod}^{org,\:mouse}$	mole/Vg/h	mAb synthesis rate in an organ in mice	For heart, kidney, spleen, and lung, $\:{K}_{prod}^{org,\:mouse}=$15.82E-21; For other organs, $\:{K}_{prod}^{org,\:mouse}=$1.07E-21	[8]
$\:{K}_{prod}^{org}$	mole/Vg/h	mAb synthesis rate in an organ in rats	*$\:{K}_{prod}^{org}={K}_{prod}^{org,\:mouse}\bullet\:SF$	***Calculated***
SF	-	A scaling factor that relates mAb synthesis rates in rats to those in mice	*-	***Estimated***
τ_org_	h	Residence time of each transit compartment of transgene expression for an organ	For liver, $\:{\tau\:}_{org}=$6.9; For heart, lung, spleen, and muscle, $\:{\tau\:}_{org}=$69.6; For other organs, $\:{\tau\:}_{org}=$30.3	[8]
$\:{P1}_{org}$,$\:{P2}_{org}$,$\:{P3}_{org}$	M	Transgene concentrations in the first, second, and third signal transduction transit compartments for mAb production in parenchymal cells of an organ	Dynamic	-
$\:{P1}_{CM}$,$\:{P2}_{CM}$,$\:{P3}_{CM}$,$\:{P1}_{SAS}$,$\:{P2}_{SAS}$,$\:{P3}_{SAS}$	M	Transgene concentrations in the first, second, and third signal transduction transit compartments for mAb production in meningeal cells in CM and SAS	Dynamic	-
$\:{E1}_{org}$,$\:{E2}_{org}$,$\:{E3}_{org}$	M	Transgene concentrations in the first, second, and third signal transduction transit compartments for mAb production in endothelial cells of an organ	Dynamic	-
$\:{E1}_{BBB}$,$\:{E2}_{BBB}$,$\:{E3}_{BBB}$,$\:{E1}_{BCSFB}$,$\:{E2}_{BCSFB}$,$\:{E3}_{BCSFB}$	M	Transgene concentrations in the first, second, and third signal transduction transit compartments for mAb production in BBB and BCSFB	Dynamic	-
$\:{K}_{sec}^{org}$	1/h	First-order secretion rate constant of intracellular produced mAb	0.022	[8]
$\:{C}_{org}^{P,\:mAb}$,$\:{C}_{org}^{E,\:mAb}$	M	mAb concentrations in cytosols of parenchymal cells and endothelial cells in an organ owing to transgene expression	Dynamic	-
$\:{C}_{CM}^{MEN,\:mAb}$,$\:{C}_{SAS}^{MEN,\:mAb}$	M	mAb concentrations in cytosols of meningeal cells in CM and SAS owing to transgene expression	Dynamic	-
$\:{C}_{BBB}^{E,\:mAb}$,$\:{C}_{BCSFB}^{E,\:mAb}$	M	mAb concentrations in cytosols of BBB and BCSFB owing to transgene expression	Dynamic	-
$\:{C}_{org}^{P,\:TargetX}$,$\:{C}_{org}^{P,\:comp}$,$\:{C}_{org}^{E,\:TargetX}$,$\:{C}_{org}^{E,\:comp}$	M	TargetX and mAb-TargetX complex concentrations in cytosols of parenchymal cells and endothelial cells in an organ owing to transgene expression (redundant)	0	***Fixed***
$\:{C}_{CM}^{MEN,\:TargetX}$,$\:{C}_{SAS}^{MEN,\:TargetX}$,$\:{C}_{CM}^{MEN,\:comp}$,$\:{C}_{SAS}^{MEN,\:comp}$	M	TargetX and mAb-TargetX complex concentrations in cytosols of meningeal cells in CM and SAS owing to transgene expression (redundant)	0	***Fixed***
$\:{C}_{BBB}^{E,\:TargetX}$,$\:{C}_{BCSFB}^{E,\:TargetX}$,$\:{C}_{BBB}^{E,\:comp}$,$\:{C}_{BCSFB}^{E,\:comp}$	M	TargetX and mAb-TargetX complex concentrations in cytosols of BBB and BCSFB owing to transgene expression (redundant)	0	***Fixed***
**anti-TargetX mAb and TargetX biodistribution**				
$\:{Q}_{Org}$,$\:{Q}_{Org}^{BC}$,$\:{J}_{Org}$	L/h	Plasma flow to, blood cell flow to, and lymph flow from an organ	-	[11]
L_lymph_	L/h	Clearance from lymph nodes	3.395	[11]
$\:{V}_{Plasma}$,$\:{V}_{BC}$,$\:{V}_{Lymph}$	L	Volumes of the central plasma, central blood cells, and lymph nodes	-	[11]
$\:{V}_{Org}^{V}$,$\:{V}_{Org}^{BC}$,$\:{V}_{Org}^{E}$,$\:{V}_{Org}^{I}$,$\:{V}_{Org}^{C}$	L	Volumes of the vascular, blood cell, endosomal, interstitial, and cellular compartments for an organ	-	[11]
$\:{C}_{Plasma}^{mAb}$,$\:{C}_{Lymph}^{mAb}$	M	mAb concentrations in the central plasma and lymph nodes	Dynamic	-
$\:{C}_{Plasma}^{TargetX}$,$\:{C}_{Lymph}^{TargetX}$	M	TargetX concentrations in central plasma and lymph nodes	Dynamic	-
$\:{C}_{Plasma}^{comp}$,$\:{C}_{Lymph}^{comp}$	M	mAb-TargetX complex concentrations in central plasma and lymph nodes	Dynamic	-
$\:{C}_{Org}^{V,\:mAb}$,$\:{C}_{org}^{E,UB,mAb}$,$\:{C}_{org}^{E,B,mAb}$,$\:{C}_{org}^{I,mAb}$	M	mAb concentrations in the vascular, endosomal (FcRn unbound and bound), and interstitial space for an organ	Dynamic	-
$\:{C}_{Org}^{V,\:TargetX}$,$\:{C}_{org}^{E,UB,TargetX}$,$\:{C}_{org}^{E,B,TargetX}$,$\:{C}_{org}^{I,TargetX}$	M	TargetX concentrations in the vascular, endosomal (FcRn unbound and bound), and interstitial space for an organ	Dynamic	-
$\:{C}_{Org}^{V,\:comp}$,$\:{C}_{org}^{E,UB,comp}$,$\:{C}_{org}^{E,B,comp}$,$\:{C}_{org}^{I,comp}$	M	mAb-TargetX complex concentrations in the vascular, endosomal (FcRn unbound and bound), and interstitial space for an organ	Dynamic	-
$$\:{C}_{BBB}^{E,B,mAb},\:\:{C}_{BBB}^{E,UB,mAb},$$$$\:{C}_{BCSFB}^{E,B,mAb}$$,$$\:{C}_{BCSFB}^{E,UB,mAb}$$	M	Concentrations of endosomal (FcRn bound and unbound) antibody in BBB and BCSFB	Dynamic	-
$\:{C}_{BBB}^{E,B,TargetX},\:\:{C}_{BBB}^{E,UB,TargetX},\:\:{C}_{BCSFB}^{E,B,TargetX}$,$\:{C}_{BCSFB}^{E,UB,TargetX}$	M	Concentrations of endosomal (FcRn bound and unbound) TargetX in BBB and BCSFB	Dynamic	-
$$\:{C}_{BBB}^{E,B,comp},$$$$\:{C}_{BBB}^{E,UB,comp}$$,$$\:{C}_{BCSFB}^{E,B,comp}$$,$$\:{C}_{BCSFB}^{E,UB,comp}$$	M	Concentrations of endosomal (FcRn bound and unbound) mAb-TargetX complex in BBB and BCSFB	Dynamic	-
$\:{C}_{Brain}^{SAS,\:mAb}$,$\:{C}_{Brain}^{LV,mAb}$,$\:{C}_{Brain}^{TFV,\:mAb}$,$\:{C}_{Brain}^{CM,mAb}$	M	mAb concentrations in SAS, LV, TFV, and CM compartments	Dynamic	-
$\:{C}_{Brain}^{SAS,\:TargetX}$,$\:{C}_{Brain}^{LV,TargetX}$,$\:{C}_{Brain}^{TFV,\:TargetX}$,$\:{C}_{Brain}^{CM,TargetX}$	M	TargetX concentrations in SAS, LV, TFV, and CM compartments	Dynamic	-
$\:{C}_{Brain}^{SAS,\:comp}$,$\:{C}_{Brain}^{LV,comp}$,$\:{C}_{Brain}^{TFV,\:comp}$,$\:{C}_{Brain}^{CM,comp}$	M	TargetX mAb-TargetX complex concentrations in SAS, LV, TFV, and CM compartments	Dynamic	-
$\:{C}_{Brain}^{PV,Art,mAb}$,$\:{C}_{Brain}^{PV,Art,TargetX}$,$\:{C}_{Brain}^{PV,Art,comp}$	M	Perivascular arterial concentrations of free mAb, TargetX, and mAb-TargetX complex	Dynamic	-
$\:{C}_{Brain}^{PV,Ven,mAb}$,$\:{C}_{Brain}^{PV,Ven,TargetX}$,$\:{C}_{Brain}^{PV,Ven,comp}$	M	Perivascular venous concentrations of free mAb, TargetX, and mAb-TargetX complex	Dynamic	-
$\:{C}_{Brain}^{P,E,mAb}$,$\:{C}_{Brain}^{P,E,TargetX}$,$\:{C}_{Brain}^{P,E,comp}$	M	Endosomal mAb, TargetX, and mAb-TargetX complex concentrations in brain parenchymal cells owing to nonspecific pinocytosis	Dynamic	-
$\:{FcRn}_{org}$	M	Unbound FcRn concentrations in the endosomes of all organs excluding brain	Dynamic	-
$\:{FcRn}_{BBB}$,$\:{FcRn}_{BCSFB}$	M	Unbound FcRn concentrations in BBB and BCSFB endosomes	Dynamic	-
FcRn_0	M	Total functional FcRn concentration in endosomal compartments	4.98E-5	[11]
$\:{\sigma\:}_{Org}^{V,mAb}$,$\:{\sigma\:}_{Org}^{V,comp}$,$\:{\sigma\:}_{Org}^{V,AAV}$	-	Vascular reflection coefficients of the mAb, the mAb-TargetX complex, and AAV for an organ	Lung: 0.95 Muscle: 0.95 Skin: 0.95 Adipose: 0.95 LI: 0.95 Kidney: 0.9 Heart: 0.9 Pancreas: 0.9 Thymus: 0.9 SI: 0.9 Liver: 0.85 Spleen: 0.85 Bone: 0.85 Others: 0.95	[11]
$\:{\sigma\:}_{Org}^{V,TargetX}$	-	Vascular reflection coefficient of TargetX	0.3116	***Calculated***
$\:{\sigma\:}_{Org}^{I}$	-	Lymphatic reflection coefficients for an organ	0.2	[11]
$\:{Diff}_{mAb}$,$\:{Diff}_{TargetX}$,$\:{Diff}_{comp}$	-	Diffusion coefficient of mAb, TargetX, and the mAb-TargetX complex	0, 24.75, 0	***Calculated***
$\:{K}_{on}^{FcRn,mAb}$,$\:{K}_{on}^{FcRn,TargetX}$,$\:{K}_{on}^{FcRn,comp}$	1/M/h	Association rate constants of FcRn binding to the antibody, TargetX, and the antibody-TargetX complex, respectively	8 × 10^8^, 0, 8 × 10^8^	[11]
$\:{K}_{off}^{FcRn,mAb}$,$\:{K}_{off}^{FcRn,TargetX}$,$\:{K}_{off}^{FcRn,comp}$	1/h	Dissociation rate constants of FcRn binding to the antibody, TargetX, and the antibody-target complex, respectively	144	[11]
$\:{K}_{on}^{mAb,TargetX}$	1/M/h	Association rate of anti-TargetX mAb binding to rat TargetX	$\:\frac{{K}_{off}^{mAb,TargetX}}{{K}_{D}^{mAb,TargetX}}$	***Calculated***
$\:{K}_{off}^{mAb,TargetX}$	1/h	Dissociation rate of anti-TargetX mAb binding to rat TargetX	-	***Estimated***
$\:{K}_{D}^{mAb,TargetX}$	M	Equilibrium dissociation constant for anti-TargetX mAb binding to rat TargetX	7.4 × 10^− 11^	***Measured***
$\:{CL}_{R}^{mAb}$,$\:{CL}_{R}^{TargetX}$,$\:{CL}_{R}^{comp}$	L/h	Renal clearance of anti-TargetX mAb, TargetX, and the antibody-target complex, respectively	0, 0.1, 0	***Calculated***
$\:{Ksyn}_{Plasma}^{TargetX}$,$\:{Ksyn}_{ISF}^{TargetX}$,$\:{Ksyn}_{LV}^{TargetX}$	M/h	Synthesis rate of endogenous TargetX in rat central plasma, brain ISF, and brain CSF (the LV compartment)	1.2 × 10^− 8^, 2.2 × 10^− 12^, 1.6 × 10^− 10^	***Calibrated***
$$\begin{array}{c}\:{Ksyn}_{Plasma}^{mAb},\:{Ksyn}_{Plasmsa}^{comp},\:{Ksyn}_{ISF}^{mAb},\\\:{Ksyn}_{ISF}^{comp},\:{Ksyn}_{CSF}^{mAb},\:{Ksyn}_{CSF}^{comp}\\\end{array}$$	M/h	Synthesis rate of anti-TargetX mAb and the antibody-target complex in central plasma, brain ISF, and brain CSF (redundant)	0	***Fixed***
FR	-	The fraction of FcRn bound antibody that recycles to the vascular space	0.715	[12]
CL_up_	L/h/L	Pinocytosis/exocytosis rate per unit endosomal volume in organs excluding brain	*0.1537	***Estimated***
CL_up,brain_	L/h/L	Pinocytosis/exocytosis rate per unit endosomal volume in brain	0.0305	[13]
CL_up,brain,cell_	L/h	Pinocytosis/exocytosis rate in brain parenchymal cells (same as$\:{Q}_{ISF}$)	1.9 × 10^− 5^	[9]
Kdeg	1/h	First-order degradation rate constant of free antibody in the endosome	*30.4863	***Estimated***
$\:{\sigma\:}_{BBB}^{V,AAV}$,$\:{\sigma\:}_{BBB}^{V,mAb}$,$\:{\sigma\:}_{BBB}^{V,\alpha\:syn}$,$\:{\sigma\:}_{BBB}^{V,comp}$	-	Vascular reflection coefficient of AAV, mAb, TargetX, and the mAb-target complex for BBB	0.9975	[9]
$\:{\sigma\:}_{BCSFB}^{V,AAV}$,$\:{\sigma\:}_{BCSFB}^{V,mAb}$,$\:{\sigma\:}_{BCSFB}^{V,TargetX}$,$\:{\sigma\:}_{BCSFB}^{V,comp}$	-	Vascular reflection coefficient of AAV, mAb, TargetX, and the mAb-target complex for BCSFB	0.9973	[13]
$\:{\sigma\:}_{CSF}^{LV-TFV,AAV}$,$\:{\sigma\:}_{CSF}^{LV-TFV,mAb}$,$\:{\sigma\:}_{CSF}^{LV-TFV,TargetX}$,$\:{\sigma\:}_{CSF}^{LV-TFV,comp}$	-	Reflection coefficient of AAV, mAb, TargetX, and the mAb-target complex for transport from LV to TFV	0.2	[9]
$$\begin{array}{c}\:{\mathrm\sigma\:}_{\mathrm{CSF}}^{\mathrm{TFV}-\mathrm{CM},\mathrm{AAV}},\:{\mathrm\sigma\:}_{\mathrm{CSF}}^{\mathrm{TFV}-\mathrm{CM},\mathrm{mAb}},\\\:{\mathrm\sigma\:}_{\mathrm{CSF}}^{\mathrm{TFV}-\mathrm{CM},\mathrm{TargetX}},\:{\mathrm\sigma\:}_{\mathrm{CSF}}^{\mathrm{TFV}-\mathrm{CM},\mathrm{comp}}\end{array}$$	-	Reflection coefficient of AAV, mAb, TargetX, and the mAb-target complex for transport from TFV to CM	0.2	[9]
$$\begin{array}{c}{\sigma\:}_{CSF}^{CM-SAS,AAV},\:{\sigma\:}_{CSF}^{CM-SAS,mAb},\\{\sigma\:}_{CSF}^{CM-SAS,TargetX},\:{\sigma\:}_{CSF}^{CM-SAS,comp}\end{array}\:\:$$	-	Reflection coefficient of AAV, mAb, TargetX, and the mAb-target complex for transport from CM to SAS	0.2	(9)
$\:{\sigma\:}_{SAS}^{I}$	-	Reflection coefficient of AAV, mAb, TargetX, and the mAb-target complex for transport from SAS to the lymph	0.2	[9]
$\:{PS}_{PV}^{TargetX}$,$\:{PS}_{PV}^{mAb}$,$\:{PS}_{PV}^{comp}$,$\:{PS}_{PV}^{AAV}$	L/h	Permeability surface area product that describes the exchange of TargetX, antibody, antibody-target complex, and AAV between the brain interstitial space and perivascular space, respectively	5.667 × 10^− 6^, 2.62 × 10^− 7^, 2.62 × 10^− 7^, 2.62 × 10^− 7^	***Supplementary Material***
$\:\frac{{SA}_{BBB}}{{SA}_{BBB}+{SA}_{BCSFB}}$	-	Fraction of BBB in total surface area of brain endothelia	0.8611	[13]
$\:\frac{{V}_{Brain}^{LV}}{{V}_{Brain}^{LV}+{V}_{Brain}^{TFV}}$	-	Fraction of LV in the total volume of ventricles	0.5	[9]
Q_ISF_	L/h	ISF formation rate	1.9 × 10^− 5^	[9]
Q_CSF_	L/h	CSF formation rate	1.32 × 10^− 4^	[9]
$\:{Q}_{CSF}^{OSC}$	L/h	Oscillatory CSF flow rate	5.04 × 10^− 5^	[9]
Q_PV_	L/h	Perivascular flow that controls influx into the periarterial space and efflux from the perivenous space	7.31 × 10^− 7^	[9]
$\:{V}_{Brain}^{BBB}$	L	Volume of the BBB endosomal compartment	9.817 × 10^− 6^	[9]
$\:{V}_{Brain}^{BCSFB}$	L	Volume of the BCSFB endosomal compartment	1.583 × 10^− 6^	[9]
$\:{V}_{Brain}^{LV}$	L	Volume of LV	7.5 × 10^− 6^	[9]
$\:{V}_{Brain}^{TFV}$	L	Volume of TFV	7.5 × 10^− 6^	[9]
$\:{V}_{Brain}^{CM}$	L	Volume of CM	1.7 × 10^− 5^	[9]
$\:{V}_{Brain}^{SAS}$	L	Volume of SAS	1.9 × 10^− 4^	[9]
$\:{V}_{Brain}^{PV}$	L	Total perivascular space volume	2.8 × 10^− 5^	[9]
$\:{V}_{Brain}^{PV,Art}$,$\:{V}_{Brain}^{PV,Ven}$	L	Perivascular artery and venous space volume	1.4 × 10^− 5^	[9]
$\:{V}_{Brain}^{MEN}$	L	Volume of meningeal cells	0.000024	***Supplementary Material***
$\:{V}_{Brain}^{P,Endo}$	L	Endosomal volume of brain parenchymal cells	3.534 × 10^− 4^	[9]

*Compared with our previous mouse AAV PBPK model [8] and rat brain antibody PBPK model [9], the following parameters are updated: (i)$\:{R}_{tot}^{BC}$, to capture AAV9 blood profiles in rats, as the mouse AAV model represented averaged profiles of AAV9 and AAV8; (ii)$\:{R}_{tot}^{Meningeal}$, which was absent in both earlier models, to account for local transduction following intra-CNS administration in rats; (iii)* SF*, not included in the mouse AAV model, to reflect lower metabolic activity in rats relative to mice; (iv)$\:{FR}_{lymph}^{AAV}$, set to 0 rather than 1 in the rat antibody model, to represent ISF-CSF exchange pathways that were not explicitly recapitulated before; (v)$\:{CL}_{up}$and$\:{K}_{deg}$estimated for anti-TargetX mAb

The mAb parameters were largely taken from the platform antibody PBPK model, and PK characteristics of the mAb-TargetX complex were assumed to be the same as the mAb. We derive essential PK parameters for TargetX, a 14 kDa protein, based on the two-pore formalism. Throughout the model, the TargetX specifically refers to the soluble monomeric form. For TargetX, the reflection coefficient ($\:{\sigma\:}_{Org}^{V,TargetX}$) was determined to be 0.3116, the diffusion coefficient ($\:{Diff}_{TargetX}$) 24.75, the renal clearance ($\:{CL}_{R}^{TargetX}$) 0.1 L/h, and the permeability-surface area product in brain perivascular space ($\:{PS}_{PV}^{TargetX}$) $\:5.667\times\:{10}^{-6}$ L/h (derivation in the Supplementary Material).

Parameters associated with AAV5 disposition were derived in a similar fashion to what has been demonstrated in our original AAV PBPK model for AAV8/9 [8]. It has been observed that 13.7% of surface bound AAV5 was internalized into airway epithelia after 1 h incubation at 37 °C [14]. According to the equation $\:{A}_{intracellular}\left(t\right)=1-{e}^{-{K}_{int}\bullet\:t}$, $\:{K}_{int}$ of AAV5 is 0.15/h. Its apparent binding affinity to COS cells was reported to be 24 pM [15], so $\:{K}_{D}^{AAV}$ of AAV5 was set to 24 pM, with $\:{K}_{off}^{AAV}$ being the same as AAV9. A study that simultaneously investigated cell surface binding of different AAV serotypes revealed that the cell surface receptor concentration for AAV5 could be more than 100-fold higher than that of AAV8 and AAV9 [16]. Accordingly, most of the tissue AAV5 receptor concentrations were set much higher than that for AAV8/9 in the model. Calibration of other AAV5 parameters was made based on the observed data (detailed in the Results section).

### Model implementation

Complete ordinary differential equations of this QSP model are provided in the Supplementary Material. Mass balance of AAV, anti-TargetX mAb, TargetX, and mAb-TargetX complex have all been confirmed to be 100%. A sequential model fitting strategy is detailed in the Results section. Model fitting was performed in R using the *Ubiquity* package (v2.0.0) [17], to obtain the maximum likelihood parameter estimates using the LSODA solver. Simulation results were cross validated in Simbiology (MATLAB 2022b, Mathworks) using the ode15s solver.

### Local sensitivity analysis

A local sensitivity analysis was performed on the final model to assess the sensitivity of various model outputs to key model parameters related to AAV transduction, transgene expression and transgene kinetics. Simulations were conducted for an ICM administration of 1E12 Vg AAV9-anti-TargetX mAb. Parameters evaluated include cellular AAV receptor concentration (blood cells $\:{R}_{org}^{BC}$, heart $\:{R}_{tot}^{Heart}$, meningeal cells $\:{R}_{tot}^{MEN}$), intracellular trafficking parameters shared by AAV receptors and AAV-receptor complex ($\:{K}_{int}$, $\:{K}_{rec}$, and $\:{K}_{trans}$), $\:{K}_{nuc}^{AAV}$, $\:{K}_{deg}^{AAV}$, transgene synthesis rate ($\:{K}_{prod}^{org}$), transgene secretion rate ($\:{K}_{sec}^{org}$), protein pinocytosis rates in all organs ($\:{CL}_{up,\:all\:org}$), and protein degradation rates in all organs ($\:{K}_{deg}$). Model outputs evaluated include plasma and tissue concentrations of AAV and anti-TargetX mAb, as well as TargetX suppression in ISF/CSF, at week 3 post-dose, as an approximation of steady state. The percentage change in these model outputs were assessed following ± 50% perturbation in selected parameters.

## Results

### Calibration of the transgene-target (mAb-TargetX) PBPK model

**Fig. 3:**
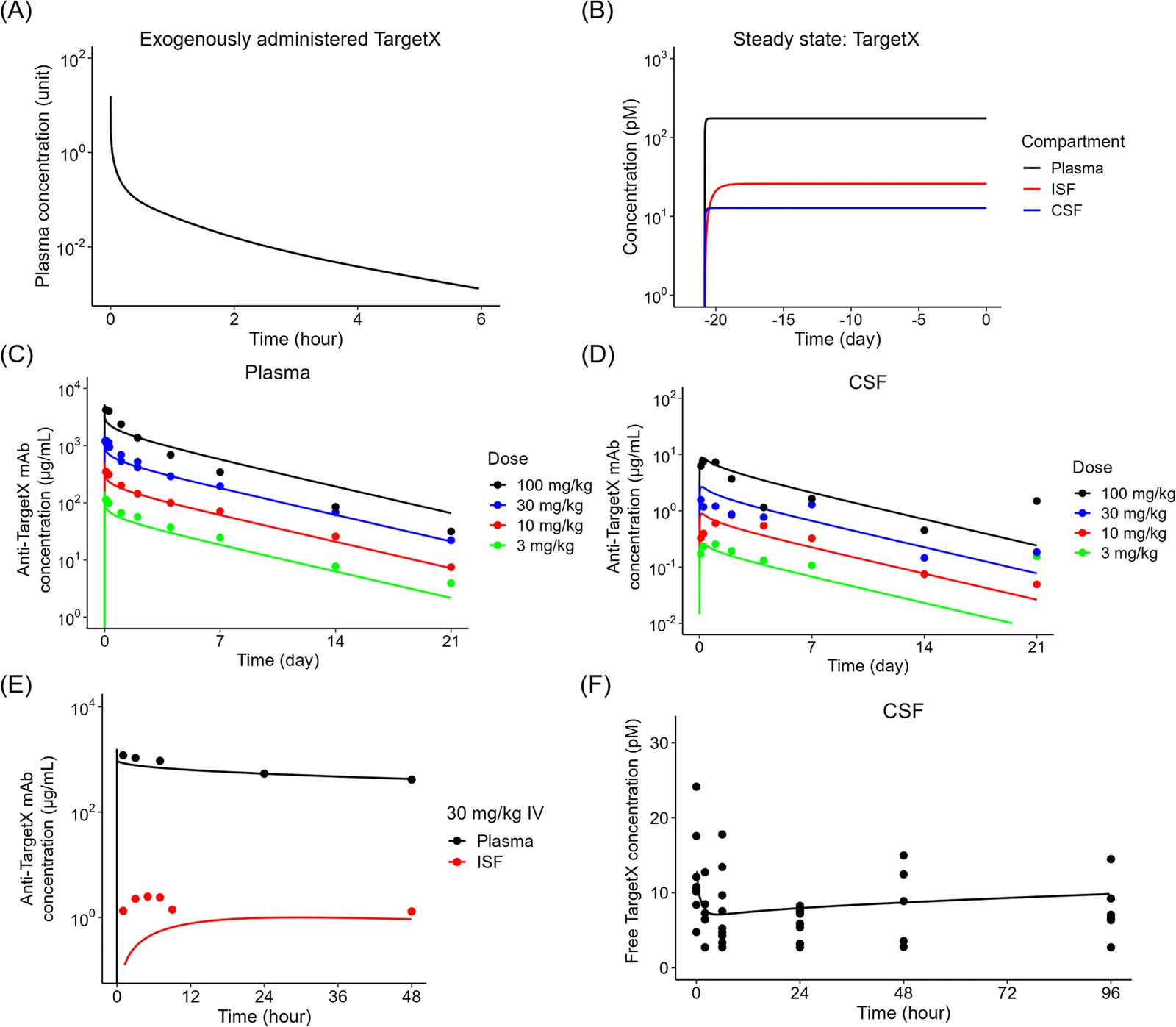
Calibration of the transgene (i.e., antibody) and target PBPK model. **(A)** Model simulated PK profile of a bolus dose of TargetX in rats. **(B)** Model simulated TargetX concentrations in plasma, ISF, and CSF at steady state prior to AAV dosing in rats. **(C)** Plasma PK upon IV administration of 3, 10, 30, and 100 mg/kg of anti-TargetX mAb in rats. **(D)** CSF PK after IV administration of 3, 10, 30, and 100 mg/kg of anti-TargetX mAb in rats. **(E)** Plasma and ISF PK after IV administration of 30 mg/kg of anti-TargetX mAb in rats. **(F)** Free TargetX concentrations in CSF in response to IV administration of 30 mg/kg anti-TargetX mAb in rats. Solid circles are observed data and solid lines are predicted typical profiles (A and B) or population fits (C, D, E, and F)

To ensure the accuracy of parameters involved in the AAV-transgene-target QSP model, the PBPK models of anti-TargetX mAb and TargetX were first calibrated using internal data. The data included plasma and CSF PK of anti-TargetX antibody, and the effect of anti-TargetX antibody administration on the turnover of TargetX (Fig. 3). The simulated PK profile of exogenously dosed TargetX had a half-life of 1.4 h (Fig. 3A), which corresponds well with the reported 1.1 h half-life of human TargetX in mice. Synthesis rates of endogenous TargetX in rats were then calibrated ($\:{Ksyn}_{Plasma}^{TargetX}=$1.2 × 10^− 8^ M/h, $\:{Ksyn}_{ISF}^{TargetX}$=2.2 × 10^− 12^ M/h, $\:{Ksyn}_{LV}^{TargetX}=$1.6 × 10^− 10^ M/h) to yield steady-state TargetX concentrations in plasma, brain ISF, and CSF of 175 pM, 26 pM, and 23 pM, respectively (Fig. 3B). These levels match the physiological ranges of TargetX in these compartments for mice, rats, nonhuman primates, or humans, as reported in the literature and measured in-house.

Plasma PK of anti-TargetX mAb following IV administration in rats at 3, 10, 30, and 100 mg/kg dose were fitted (Fig. 3C), enabling parameter estimates of $\:{CL}_{up}$ and $\:{K}_{deg}$ of anti-TargetX mAb (Table 2). Simulated CSF and ISF PK profiles of anti-TargetX mAb at these dose levels are shown in Fig. 3D and E. In general, PK data of anti-TargetX mAb was captured reasonably well. Underpredictions of ISF concentrations were observed in the first 12 h, which may be attributed to local damage in the brain by the microdialysis probe, or certain blood-to-ISF pathway that has not been explicitly characterized in the model yet. Free TargetX concentrations in CSF following IV dosing of 30 mg/kg anti-TargetX mAb were fitted (Fig. 3F), with $\:{K}_{off}^{mAb,TargetX}$ value estimated to be 0.00427/h (Table 2), and assumption that the affinity of anti-TargetX mAb binding to rat TargetX is the same as its binding to the human counterpart (74 pM, Table 1).

**Table 2:** Parameter estimates and associated uncertainties from the AAV9-mAb-TargetX QSP model fitting

Parameter	Unit	Final estimate	CV%
CL_up_	L/h/L	0.154	3.32
K_deg_	1/h	30.5	6.61
$\:{K}_{off}^{mAb,TargetX}$	1/h	0.00427	48.64
$\:{R}_{tot}^{BC}$	copy/L	7.12E11	< 0.01
$\:{R}_{tot}^{MEN}$	copy/L	3.8 E15	17.47
SF	-	0.11	5.32

## Calibration of the AAV-transgene (AAV9-anti-TargetX mAb) PBPK model

First, the previously developed AAV8/9-mAb PBPK model was directly integrated with the brain submodel published by Wu et al. (i.e., Fig. 2 without the highlighted structure) to characterize the PK dataset. However, the following deviations were observed, and hence respective strategies were considered. First, steady-state blood concentrations of AAV9 were generally overpredicted, which was solved by decreasing AAV receptor concentration on blood cells. Second, produced antibody concentrations were largely overpredicted, indicating that the antibody production rate ($\:{K}_{prod}^{org}$) should be smaller than that used in the mouse AAV model ($\:{K}_{prod}^{org,\:mouse}$). This can be attributed to the fact that higher species have lower metabolic activity for transgene expression. Third, CSF mAb concentrations were greatly underpredicted after local injections of AAV9, as corroborated by 10-100-fold higher CSF/plasma mAb concentration ratios following ICM infusion of AAV9 relative to the IV route (~ 2% vs. ~0.2%). This strongly indicates that cells surrounding CSF CM and SAS compartments (i.e., meningeal cells) should be added in the model to account for local AAV transduction and mAb secretion into CSF. Accordingly, the AAV9-mAb PBPK model was calibrated as follows. First, AAV9 biodistribution data following IV administration were fitted to obtain $\:{R}_{tot}^{BC}$ estimates of 7.12E11 copy/L, which was fixed afterwards. Next, anti-TargetX mAb biodistribution data following IV dosing of AAV9 were fitted to estimate the ubiquitous * SF*, a scaling factor that relates mAb synthesis rates in rats to those in mice, which was also fixed later (* SF* estimated to be 0.11, Table 2). Lastly, AAV9 and mAb biodistribution data following all the local injection routes were fitted simultaneously to gain the estimate of $\:{R}_{tot}^{MEN}$. $\:{R}_{tot}^{MEN}$ was estimated to be 3.8E15 copy/L, a receptor density similar to that in liver, indicating strong retention of AAV9 by these cells in the brain.

**Fig. 4:**
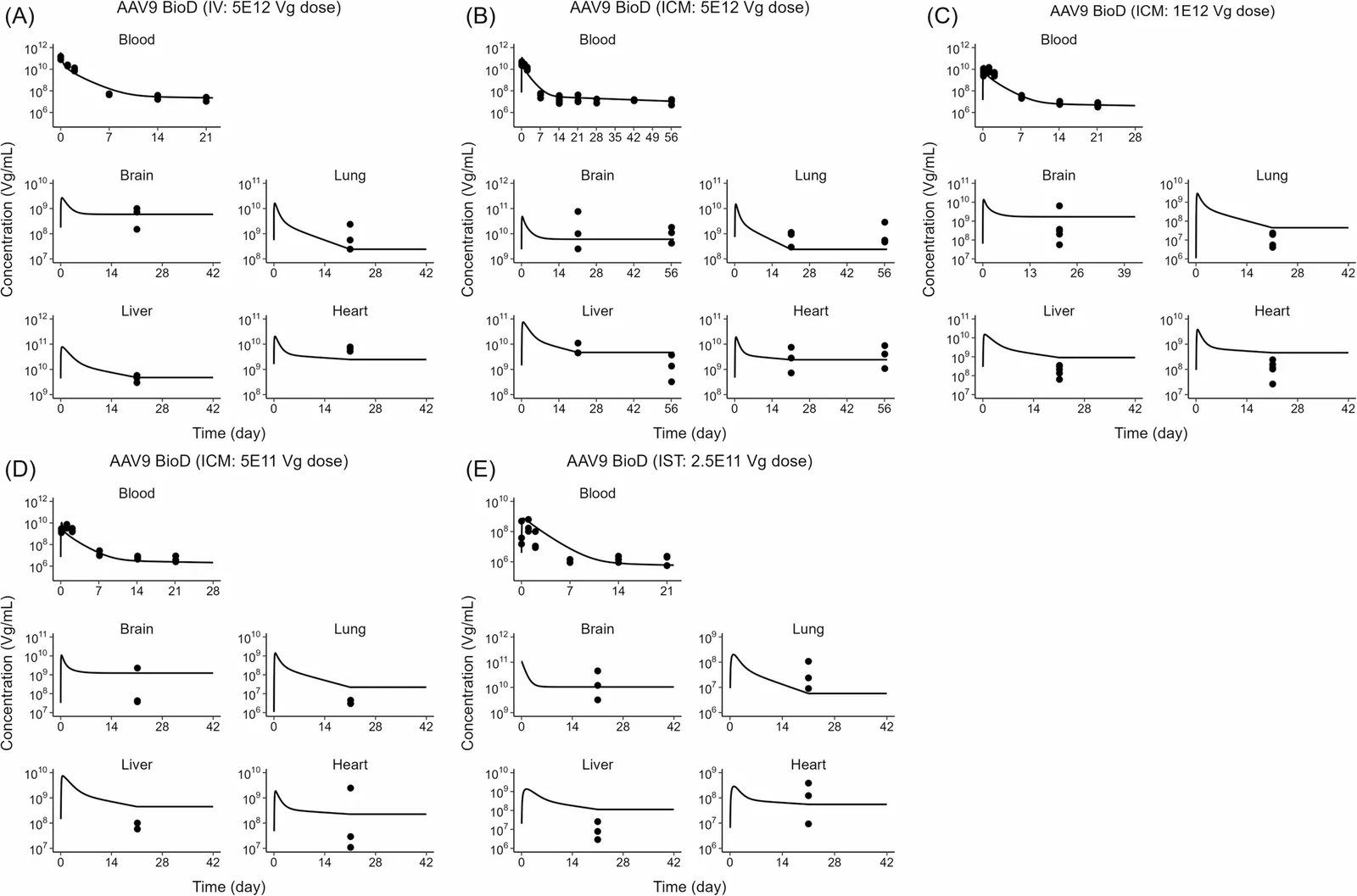
AAV9 vector distribution in blood, brain, lung, liver, and heart following:** (A)** IV dosing of 5E12 Vg AAV9-anti-TargetX mAb, **(B)** ICM dosing of 5E12 Vg AAV9-anti-TargetX mAb, **(C)** ICM dosing of 1E12 Vg AAV9-anti-TargetX mAb and AAV9-control mAb, **(D)** ICM dosing of 5E11 Vg AAV9, or **(E)** IST dosing of 2.5E11 Vg AAV9-anti-TargetX mAb. Solid dots are observed data and solid lines are population fits

**Fig. 5:**
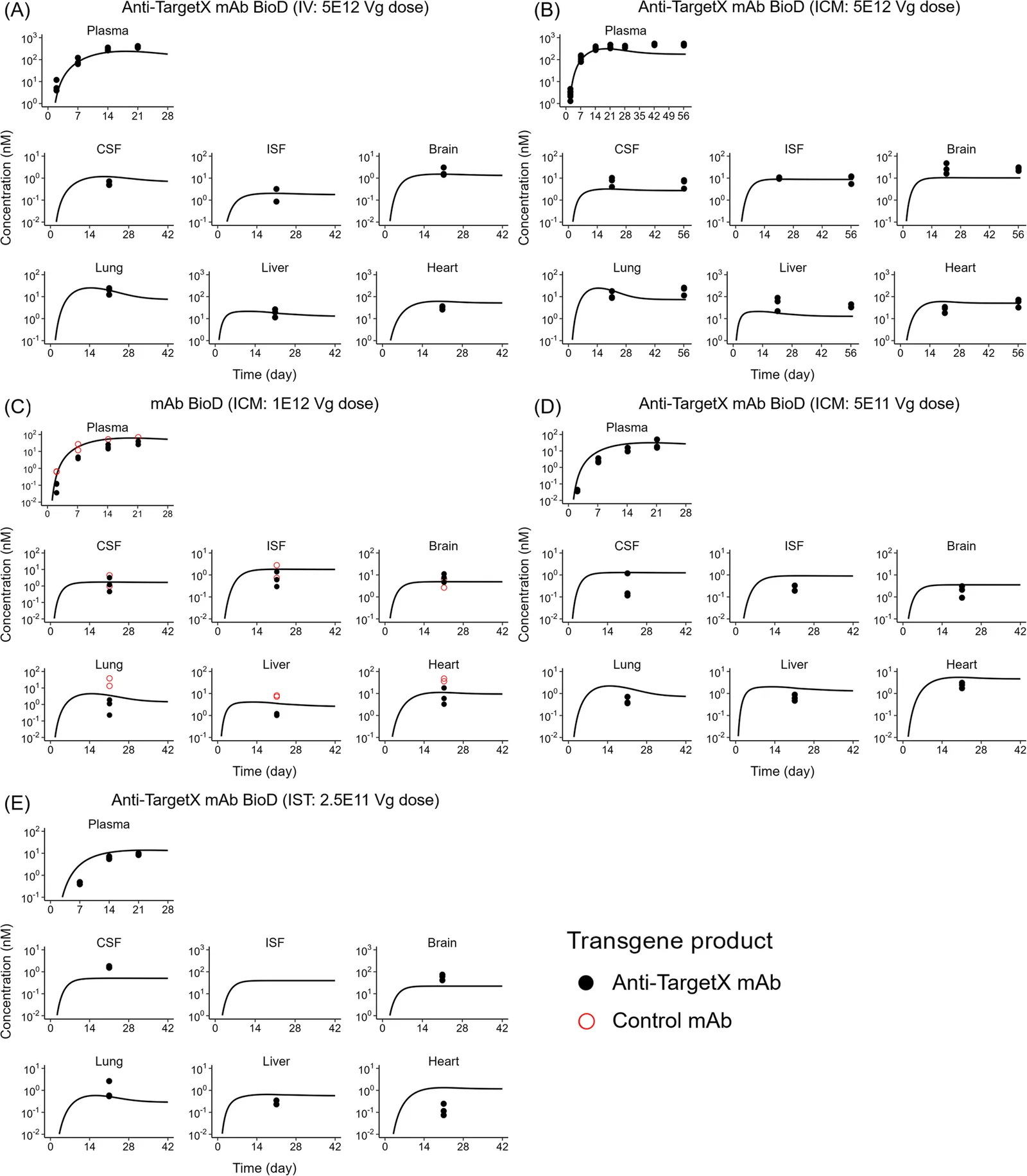
Anti-TargetX mAb distribution in plasma, CSF, ISF, brain, lung, liver, and heart after: **(A)** IV dosing of 5E12 Vg AAV9, **(B)** ICM dosing of 5E12 Vg AAV9, **(C)** ICM dosing of 1E12 Vg AAV9, **(D)** ICM dosing of 5E11 Vg AAV9, and **(E)** IST dosing of 2.5E11 Vg AAV9. Control mAb biodistribution after ICM dosing of 1E12 Vg AAV9 dosing is also shown in (C) as open red circles. Circles are observed data and solid lines are population fits

Vector and transgene PK predictions of the final unified model are shown in Figs. 4 and 5, respectively. Generally, these PK profiles were captured well without systematic bias. Deviations from observed data were noted in several scenarios. First, for AAV9 dosed via ICM at 1E12 Vg, vector disposition in lung, liver, and heart was overpredicted, yet the corresponding transgene PK profiles did not show the same trend (Figs. 4C and 5C). Meanwhile, measured control mAb concentrations are consistently higher than those of anti-TargetX mAb in most metrices (Fig. 5C), which indicates that binding to TargetX may cause steric hindrance to the secondary antibody detecting anti-TargetX mAb in ELISA, leading to underestimated concentrations. Alternatively, the two mAbs may have different protein synthesis rates. Second, vector blood PK profiles from the IST route at early time points were overestimated (Fig. 4E), perhaps because some bioavailability mechanisms were not properly incorporated in the model. But since brain disposition was well captured, and only one low dose was tested via the IST route, it is difficult to further improve the fit. Last, anti-TargetX mAb plasma PK beyond 3 weeks was underestimated (Fig. 5B). The original PBPK model was developed to depict the slightly decreased plasma transgene expression after week 3 as observed in mice and many literature examples, yet this pattern does not correspond to the observations in the 5E12 Vg AAV9-anti-TargetX mAb ICM group.

### Calibration of the AAV5 PBPK model

As time-concentration profiles of AAV5 in tissues were not obtained in the present study as what was obtained for AAV8/9 in our previous investigation [8], it was challenging to derive AAV5 related parameters. Nevertheless, to ensure that AAV5 parameters fall in biologically reasonable ranges, we derived a few of them ($\:{K}_{D}^{AAV}$, $\:{K}_{int}$, $\:{K}_{rec}$, $\:{K}_{trans}$) based on literature in vitro data and manually adjusted the rest ($\:{R}_{tot}^{org}$, $\:{K}_{deg}^{AAV}$, $\:{K}_{nuc}^{AAV}$), as shown in Table 3. Parameter values associated with transgene expression and secretion were kept the same as those used in the AAV9 model, because the single-stranded DNA transgene adopted by the AAV5 was the same as used in the AAV9.

**Table 3:** AAV5 parameter values that are different from those for AAV9 in the QSP model

Parameter	Unit	Value	Rationale
$\:{R}_{tot}^{org}$	copy/L	Liver: 3.2E15 Adipose: 6.0E15 Heart: 2.5E15 Lung: 1.7E16 Spleen: 6.0E15 Muscle: 6.0E15 Kidney: 6.0E15 Skin: 6.0E15 Pancreas: 6.0E15 Brain: 2.0E15 Bone: 6.0E15 Blood cells: 7.12E11 Brain meningeal: 2.0E16 Others: 6.0E15	***Calibrated***
			***Calculated***
$\:{K}_{on}^{AAV}$	L/copy/h	6.5E-15	$\:\frac{{K}_{off}^{AAV}}{{K}_{D}^{AAV}}$
$\:{K}_{D}^{AAV}$	copy/L	1.44E13 (Equivalent to 24 pM)	[15]
$\:{K}_{deg}^{AAV}$	1/h	5.0	***Calibrated***
$\:{K}_{int}$, $\:{K}_{rec}$, $\:{K}_{trans}$	1/h	0.15	[14]
$\:{K}_{nuc}^{AAV}$	1/h	0.01	***Calibrated***

**Fig. 6:**
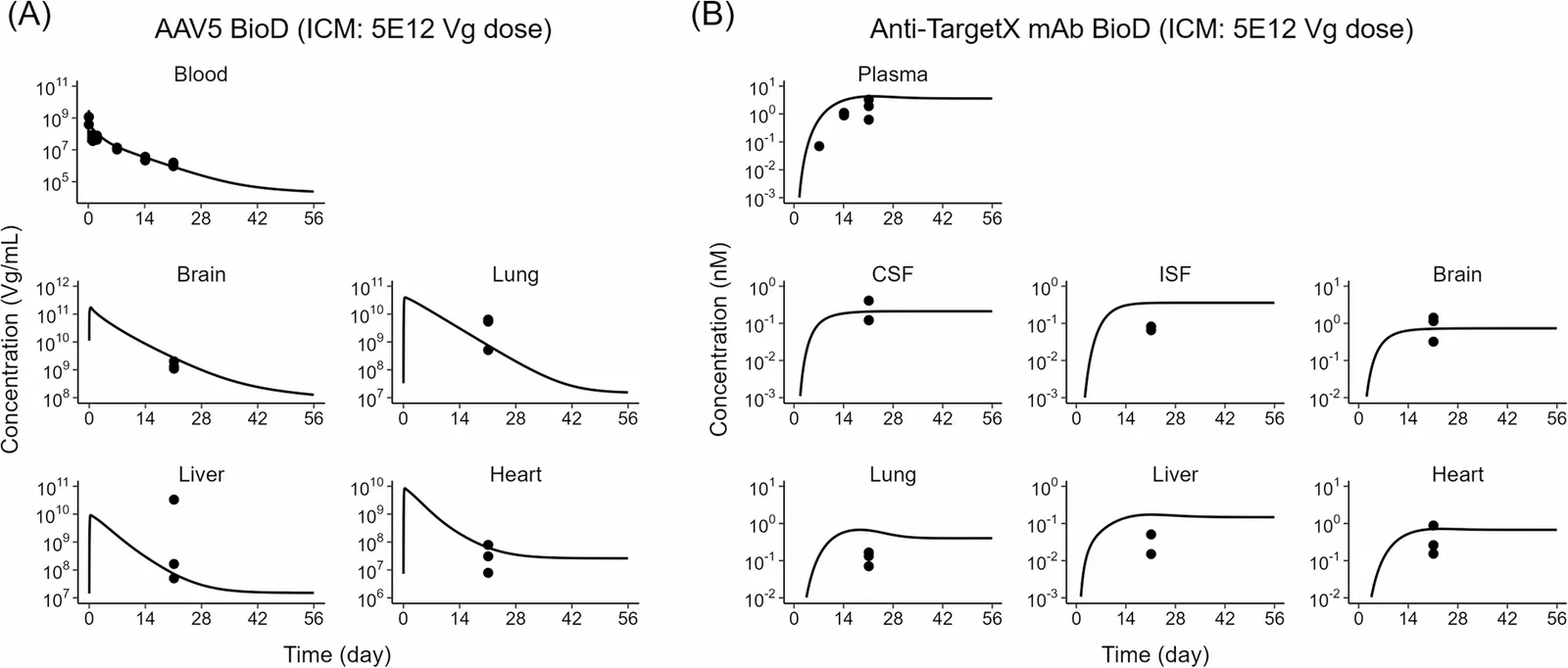
AAV5 and anti-TargetX mAb biodistribution following ICM dosing of 5E12 Vg AAV5-anti-TargetX mAb in rats. Solid circles are observed data and solid lines are model predicted typical profiles

A very intriguing phenomenon was noticed that while AAV5 vector concentrations in blood and organs at week 3 were very similar to those for AAV9 following ICM dosing at 5E12 Vg, anti-TargetX mAb expression level was almost 200-fold lower (Fig. 6 vs. Figures 4B and 5B). Therefore, we hypothesized that AAV5 DNA concentrations measured at week 3 mostly were not functional dsDNA in the nucleus driving transgene expression, but rather the inverted terminal repeat (ITR) plasmid form still undergoing intracellular processing. It can be further deduced that the steady state has not been reached at the final time point where data were collected, and that DNA concentrations will be lost beyond week 3. Accordingly, $\:{K}_{nuc}^{AAV}\:$of AAV5 needed to be extremely slow compared to AAV9 (Table 3). Figure 6 displays model predictions overlaid with observations following ICM administration of 5E12 Vg AAV5-anti-TargetX mAb. AAV5 vector PK data were captured reasonably well, whereas slight overpredictions of plasma, ISF, lung, and liver anti-TargetX mAb PK data were observed. Nevertheless, the model performance was deemed acceptable given the limited data.

### Prediction of target engagement for different AAV regimens

**Fig. 7:**
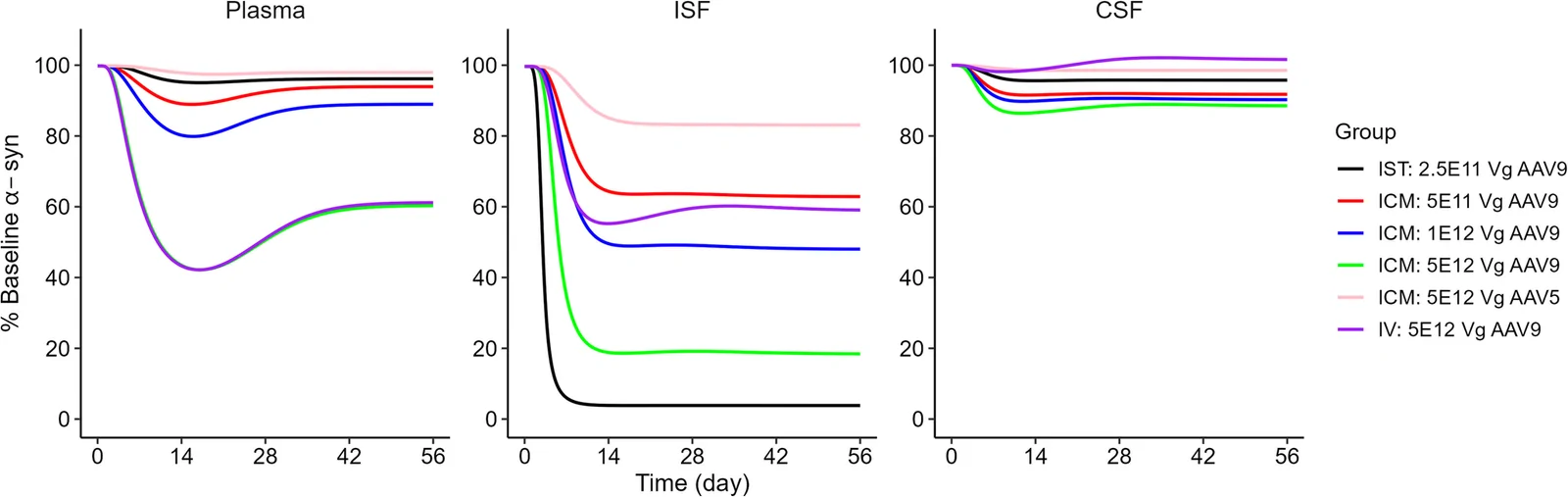
Model-predicted free TargetX concentrations relative to baseline values in plasma, ISF, and CSF following the dosing regimens tested in the present study

While not experimentally measured, free TargetX concentrations relative to baselines in plasma, ISF, and CSF were predicted by the AAV-transgene-target QSP model (Fig. 7). As anticipated, direct injections of AAV9 in the compartments can lead to the strongest suppression of TargetX in respective compartments. At similar dose levels, IV, IST, and ICM administration of AAV9 can suppress TargetX to the greatest extent in plasma, ISF, and CSF, respectively. We tested 3 dose levels for the ICM route (5E11, 1E12, and 5E12 Vg), and they demonstrate dose-dependent target engagement. ICM dosing displays perfect bioavailability and local targeting, as inferred from similar TargetX suppression in plasma and much better TargetX depletion in CSF and ISF, compared to IV dosing at 5E12 Vg. Additionally, AAV5 exhibits the worst efficacy among all the groups tested, even at a high dose level given ICM. Even at the lowest dose, IST administration was projected to lead to most suppression of the target in the ISF compartment, which is typically the site-of-action for most CNS targeted antibodies.

### Sensitivity analysis of the AAV9-anti-targetX mAb PBPK model

**Fig. 8:**
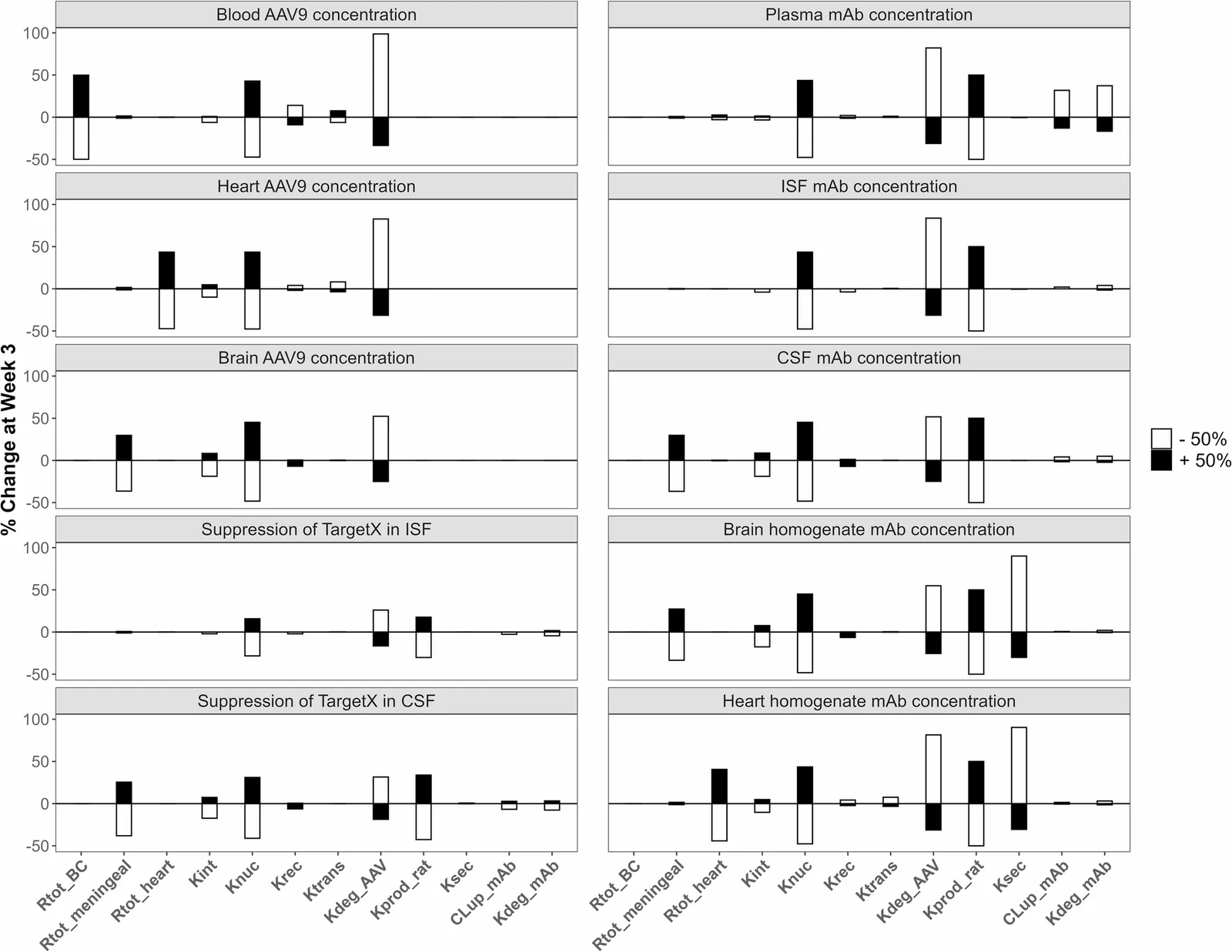
Local sensitivity analysis of parameters in the QSP model. The bars show percentage changes in blood/heart/brain AAV concentrations, plasma/brain/heart/ISF/CSF anti-TargetX mAb concentrations, and suppression of TargetX in ISF/CSF at steady state, in response to ± 50% perturbations in model parameters, following ICM dosing of 1E12 Vg AAV9-anti-TargetX mAb in rats. The higher the absolute value of percent changes, the higher the sensitivity of the parameter. Positive and negative values for percent change of TargetX suppression indicate stronger and weaker suppression effects of TargetX

To evaluate the relative importance and identifiability of model parameters, a local sensitivity analysis was conducted. Figure 8 shows the percentage changes in steady-state plasma and tissue concentrations of AAV and anti-TargetX mAb, as well as TargetX depletion in ISF/CSF, following ± 50% perturbations in selected parameters under an ICM administration of 1E12 Vg AAV9-anti-TargetX mAb.

Among AAV receptor concentrations, $\:{R}_{org}^{BC}$ has the largest influence on blood AAV concentration; however, because AAV in blood cells does not contribute to transgene expression, its effect on transgene PK and pharmacodynamics is minimal. Tissue-specific AAV receptor densities primarily affect local AAV and transgene distribution with minimal impact on systemic distribution. For example, increased $\:{R}_{tot}^{Heart}$ elevates AAV and anti-TargetX-mAb levels in heart; whereas increased $\:{R}_{tot}^{MEN}$ enhances AAV transduction and mAb production in brain, leading to stronger suppression of TargetX in CSF. Given the CSF localization of meningeal cells, $\:{R}_{tot}^{MEN}$has negligible impact on ISF transgene levels.

AAV intracellular processing parameters exhibited varied sensitivities: (1) The trafficking parameters $\:{K}_{int}$, $\:{K}_{rec}$, and $\:{K}_{trans}$ have negligible to modest effects on AAV and transgene distribution. This may be attributed to two factors. First, these rates apply to both AAV receptors and AAV-receptor complexes, so changes in trafficking can alter cellular distribution of receptors prior to dosing, when cellular (membrane + intracellular) receptor concentrations are kept unchanged. For example, although greater $\:{K}_{int}$ accelerates AAV internalization, it also reduces the number of AAV receptors available on cell membrane for binding. Second, ICM administration bypasses BBB and BCSFB transcytosis, reducing the relevance of these trafficking steps compared to IV dosing (2). In contrast, higher $\:{K}_{nuc}^{AAV}$ and lower $\:{K}_{deg}^{AAV}$ markedly increase AAV accumulation and transgene expression ubiquitously, resulting in improved TargetX depletion.

For transgene-related processes, increases in $\:{K}_{prod}^{org}$ most substantially enhances mAb expression ubiquitously and TargetX depletion. Reduced $\:{K}_{sec}^{org}$ increases tissue intracellular transgene accumulation (reflected in homogenate concentrations) but has minimal effect on systemic mAb levels at steady state. $\:{CL}_{up,\:all\:org}$ and $\:{K}_{deg}$ influence systemic mAb concentrations but has limited impact within brain compartments, likely due to the dominant contribution of meningeal cells to CSF mAb production that outweighs local elimination of mAb.

Overall, the sensitivity analysis reveals biologically interpretable results and reasonable identifiability for most parameters. The results underscore the importance of collecting relevant data (e.g., different administration routes, both plasma and tissue PK profiles) to inform different parameters, and justify the strategy to sequentially estimate those sensitive parameters. Few correlated parameters, such as $\:{K}_{nuc}^{AAV}$ and $\:{K}_{deg}^{AAV}$, demonstrate that some processes are unidentifiable by our data and therefore should be fixed to literature-derived values, as done in our previous mouse AAV model. Several manually adjusted parameters with higher uncertainty (e.g., $\:{K}_{rec}$, and $\:{K}_{trans}$) show low sensitivity under ICM dosing, further supporting the robustness of the modeling conclusions.

## Discussion

To date, there are no disease-modifying cures for Parkinson’s disease, and only symptomatic treatments are available to mitigate motor and non-motor symptoms [18]. As such, the idea of targeting a promising antigen (such as TargetX) has been pursued with the aim of halting or reversing neurodegeneration. Many mAbs have been developed but failed to yield satisfactory endpoints in clinical trials, partly due to insufficient mAb delivery to the brain. Meanwhile, the past decade has witnessed a surge of AAV development, owing to the vectors’ ability to target nondividing cells in various organs such as the brain. More than 20 AAV-mediated gene therapy clinical trials are underway for neurodegenerative or neuromuscular diseases, many of which apply IV, ICM, IST, or lumbar intrathecal administration routes [19]. Therefore, utilizing AAVs to deliver an antibody gene to the brain appears to be a viable approach.

However, it remains a big hurdle to project the first-in-human dose when moving AAV programs to the clinic. Although model-informed drug development (MIDD) has been proposed for gene therapy for a long time [20, 21], very limited progress has been made and is yet to be validated. We recently developed an AAV PBPK model based on mouse data, representing a crucial step toward MIDD of AAV therapies [8]. The present follow-up investigation was performed with multiple purposes: (1) to examine if the developed mouse AAV9-mAb PBPK model can be translated to rats; (2) to expand and enhance the PBPK model to account for brain local injections; (3) to investigate if the AAV model, which was initially developed based on AAV9 data, can serve as a generalizable platform to describe another serotype, AAV5, albeit with different parameterization; and (4) to determine the best dosing regimen of AAV therapies for treating neurological disorders. We have reasonably achieved these primary goals in the study, as discussed below.

Using a stepwise model fitting strategy, parameters associated with anti-TargetX mAb distribution, anti-TargetX mAb and TargetX binding kinetics, AAV9 receptor densities on blood cells and brain meningeal cells, and the transgene expression strength in rats were estimated with good precision. Interestingly, AAV9 constructs encoding a mAb (AAV9-anti-TargetX mAb in rats from the present study vs. AAV9-trastuzumab in mice from our previous study) exhibited very consistent PK features between mice and rats, as characterized by the PBPK model. The organ-dependent AAV9 receptor densities derived based on mouse data also seem to apply to rats, at least for brain, lung, liver, and heart; suggesting that AAV9 tropisms are highly similar in these two species. Parameters related to the transgene DNA cassette design, including the transgene expression delays ($\:{\tau\:}_{org}$), antibody secretion rate ($\:{K}_{sec}^{org}$), and transgene expression strength in different organs were found to be the same in the two species. As expected, the average transgene expression efficiency decreases by almost 10-fold in rats relative to mice (* SF* of 0.11).

On the basis of our most recent brain mAb PBPK model [9], additional features were added to accommodate the findings from the brain injection data of AAVs. A meningeal cell structure was supplemented in CM and SAS spaces, without which high CSF transgene expression following ICM dosing of AAV cannot be captured. High CSF mAb concentrations were also observed following IST dosing of AAV9, because of which we set $\:{FR}_{lymph}^{AAV}$ as 0 instead of 1 in the original model [9]. This does not necessarily indicate that all the AAVs in the perivenous space actually flows to SAS, but rather, it is an approximation of some transcytosis or flow exchange pathways between ISF and CSF not explicitly recapitulated in the current model, which facilitates transport of AAVs from ISF to CSF. Moreover, AAV9 and AAV5 are capable of transducing cells widely dispersed throughout the brain, including neurons, astrocytes, oligodendrocytes, and others [22–24], and we did not separate out cell types in the parenchymal cell pool.

One key question that the study aims to address is what administration route of which AAV serotype at what dose level is optimal to achieve maximum suppression of TargetX at the site-of-action. Owing to its transcytosis capability across the BBB [25], AAV9 has been widely tested to transduce the brain following systemic administration [26–28]. However, IV dosing of AAV9 still results in a very low fraction of the vector penetrating the BBB with excessive peripheral tissue distribution [29–31]. Accordingly, given the model simulations, AAV9-anti-TargetX mAb administered via the IV route at a high dose (5E12 Vg) showed the most prominent elimination of free TargetX in central plasma, but only mediocre inhibition of TargetX in ISF and the worst efficacy in CSF. It can be concluded that if this noninvasive route has to be chosen, a much higher dose is warranted, which will exacerbate systemic toxicity. Therefore, IV administration of AAV9-anti-TargetX mAb does not appear to be an attractive therapeutic option. Compared to the IV route, the same high dose level (5E12 Vg) of AAV9-anti-TargetX mAb given via ICM not only achieved similar TargetX inhibition in systemic circulation but also eliminated TargetX in ISF and CSF very effectively. Considering the observed dose-dependent pharmacological effect, a medium dose level (1E12 Vg) of AAV9-anti-TargetX mAb administered via ICM may be recommended, as it allowed satisfactory TargetX clearance from the brain while minimizing toxicity originating from excessive removal of TargetX in circulation (Fig. 7). The IST injection stands out among all the administration routes in terms of the low dosage needed, high local transgene expression, and limited peripheral transduction, consistent with our previous finding [32]. At the lowest dose among all (2.5E11 Vg), IST administration of AAV9-anti-TargetX mAb appeared to lead to remarkable exclusion of TargetX from ISF and CSF without influencing systemic TargetX significantly. However, when performing IST administration, ISF sample is not sufficient for quantitative measurements to validate the model predicted high anti-TargetX mAb concentrations in ISF. Therefore, to support the notion that the IST route is optimal, further studies are warranted to determine if the improved pharmacodynamic benefits outweigh side effects related to this surgical procedure [33]. Additionally, we tested another neurotropic serotype, AAV5, albeit only at the high dose (5E12 Vg) via ICM administration. AAV5-anti-TargetX mAb exhibited weaker transgene expression overall and hence inferior predicted TargetX elimination than even the low dose (5E11 Vg) of AAV9 given via ICM, and therefore should not be further pursued for this application. The sensitivity analysis of TargetX suppression at the site of action suggests opportunities to further improve efficacy with ICM dosing of AAV9. Vectors engineered with greater dsDNA formation rate and slower cytosolic degradation and gene cassettes designed for higher transgene expression could potentially enhance TargetX suppression.

Similarly to our observation, lower transgene expression in vivo following administration of AAV5 compared to AAV9 has also been reported before [34–38]. However, the phenomenon became even more elusive when we noticed that AAV5 (5E12 Vg via ICM) biodistribution at week 3 was almost the same as that of AAV9 (5E12 Vg via ICM), yet the transgene expression throughout the body was still around 200-fold lower in the former situation. Regarding the fact that comparable AAV DNA concentrations led to a much lower level of transgene expression for AAV5 relative to AAV9, we raised one hypothesis: in contrast to AAV9, the AAV5 DNA concentrations measured at week 3 mostly may not reflect functional dsDNA that drives protein expression, but the intermediate form of DNA under intracellular processing. This hypothesis was numerically reflected in a $\:{K}_{nuc}^{AAV}$ of 0.01/h, which is much smaller than $\:{K}_{nuc}^{AAV}$ of AAV9. This is corroborated by a molecular investigation revealing that circular full-length transgene DNA could not be detected at 24 h postdosing of AAV5 but was present at 1 week, which increased gradually over 8 weeks [39]. Considering that $\:{K}_{nuc}^{AAV}$ is a hybrid parameter describing the net rate of dsDNA formation from capsid-protected vector genome in endosome, we speculate that the rate-limiting step of AAV5 transduction is perhaps endosomal escape, nuclear translocation, or second-stranded DNA synthesis.

The current model was developed using data generated in healthy rats, in which endogenous TargetX exists predominantly in the soluble monomeric form rather than as pathological aggregates. As such, aggregated TargetX species were not explicitly represented in the model, and soluble TargetX was used as a pharmacodynamic surrogate. Mechanistically, monomeric TargetX serves as the precursor pool for aggregation, and antibody-mediated sequestration of soluble TargetX is assumed to reduce availability of monomeric species that can form aggregates. Therefore, sustained depletion of TargetX is expected to translate into reduced aggregation and potentially slower disease progression. This assumption needs to be evaluated in the future; however, the omission of aggregated TargetX should not affect comparisons across AAV serotypes, routes of administration, or dose levels, which are the primary objective of this work. Another limitation is the lack of an external validation dataset, as all the present data were critical for model calibration. Validation datasets involving alternative dosing regimens or administration routes will be important for confirming the model’s performance.

The next step of the research presented here is to further apply this QSP model to evaluate potential of several strategies to improve the therapeutic outcome of AAV therapies targeting CNS antigen (e.g., TargetX), such as enhancing BBB crossing capability of AAV9 via attaching shuttle peptides [40] and allowing brain-specific transgene expression [5]. This modeling framework also provides a foundation for translational applications in nonhuman primates and humans. Since the current model was developed based on rat physiology, in addition to changes in species-specific physiological parameters, disease-related parameter changes, such as BBB permeability and brain uptake rates, as well as disease-related processes, such as TargetX aggregation kinetics and altered target turnover, would need to be incorporated. With these considerations, the PBPK/QSP framework could help explore appropriate AAV serotypes, doses, and routes of administration to improve clinical outcomes.

## Conclusion

Here we have evolved platform AAV PBPK model to characterize systemic and tissue disposition of both the virus and the transgene product following intravenous and local brain injections of AAV9 and AAV5 encoding anti-TargetX mAb in rats. This model has demonstrated its potential in capturing cross-species, cross-serotypes, and cross-routes of administration data. Based on model-predicted TargetX engagement, AAV5 and IV administration of AAV9 represented the least promising therapeutic options. We conclude that IST and ICM administrations of AAV9 can help achieve therapeutic CSF and ISF exposures of antibody and minimize pathogenic target levels at the site-of-action.

## Supplementary Information

Below is the link to the electronic supplementary material.

Supplementary Material 1

## Data Availability

No datasets were generated or analysed during the current study.
