# Supplementary material for: QSP model for AAV-mediated antibody delivery in rat brain
Source: J Pharmacokinet Pharmacodyn. 2026 Jul 18;53(5):41. doi: 10.1007/s10928-026-10051-w (PMC13380586; doi:10.1007/s10928-026-10051-w)
Supplement: Supplementary file 1 — Supplementary Material 1 [file 10928_2026_10051_MOESM1_ESM.pdf]

Supplemental Material

QSP Model for AAV-Mediated Antibody Delivery in Rat Brain

Shufang Liu[1], Ekram Ahmed Chowdhury[1], Shengjia Wu[1], Guy Meno-Tetang[2], Dhaval K. Shah[1,*]

[1] Department of Pharmaceutical Sciences, School of Pharmacy and Pharmaceutical Sciences, The State University of New York at Buffalo, Buffalo, NY

[2] Neuroscience, BioPharmaceuticals R&D, AstraZeneca, Cambridge, UK

[*]Corresponding author:
Dhaval K. Shah, PhD
Department of Pharmaceutical Sciences
455 Pharmacy Building, School of Pharmacy and Pharmaceutical Sciences
University at Buffalo, The State University of New York
Buffalo, New York 14214-8033
Telephone: 716-645-4819
E-mail: dshah4@buffalo.edu

QSP Model Parameters

We derived essential PK parameters for TargetX, a 14 kDa protein, based on the two-pore formalism.

The reflection coefficients of TargetX for the large pore (σ_L) and the small pore (σ_S) are:

$$\sigma_L = 0.000035 \times MW^{0.717} = 0.000035 \times 14000^{0.717} = 0.03287319 \quad (1)$$

$$\sigma_S = 1 - 0.8489 \times e^{-0.00004 \times MW} = 1 - 0.8489 \times e^{-0.00004 \times 14000} = 0.5151006 \quad (2)$$

Where MW is the molecular weight of TargetX in Dalton.

The net lymphatic flow through large (J_L) and small (J_S) pores are:

$$J_L = \alpha_L \cdot J + J_{L,iso} = (0.042 + 0.38) \times J = 0.422\, J \quad (3)$$

$$J_S = \alpha_S \cdot J + J_{S,iso} = (0.958 - 0.38) \times J = 0.578\, J \quad (4)$$

Where α_L and α_S are fractions of hydraulic conductance attributed to the large and the small pores, J is the net lymphatic flow of an organ, and $J_{L,iso}$ and $J_{S,iso}$ are isogravimetric flow through large and small pores.

As the current PBPK model is based on one-pore theory, the equivalent convective transport can be converted from the two-pore calculation:

$$J \cdot (1 - \sigma) = J_L(1 - \sigma_L) + J_S(1 - \sigma_S) \quad (5)$$

Which reveals the reflection coefficient of TargetX (σ) to be 0.3116.

Equations (6) to (11) derive diffusive transport of TargetX extravasation.

$$\frac{A_L}{A_{oL}} = 0.3429 \times e^{-0.00012175 \times MW} + 0.6571 \times e^{-0.00000421 \times MW} = 0.6818 \quad (6)$$

$$\frac{A_S}{A_{oS}} = 0.2352 \times e^{-0.00008295 \times MW} + 0.7767 \times e^{-0.00053095 \times MW} = 0.074 \quad (7)$$

Where $\frac{A_L}{A_{oL}}$ and $\frac{A_S}{A_{oS}}$ are the fractional accessible cross-sectional pore area of large and small pores, respectively.

Permeability-surface area products through the large pore (PS_L) and the small pore (PS_S) are:

$$PS_L = X_P \cdot \frac{1}{a_e} \cdot \frac{A_L}{A_{oL}} \cdot \frac{\alpha_L \cdot J}{{r_L}^2} = 13197 \times \frac{1}{1.92465} \times 0.6818 \times \frac{0.042}{22.85^2} \times J = 0.376\,J \quad (8)$$

$$PS_S = X_P \cdot \frac{1}{a_e} \cdot \frac{A_S}{A_{oS}} \cdot \frac{\alpha_S \cdot J}{{r_S}^2} = 13197 \times \frac{1}{1.92465} \times 0.074 \times \frac{0.958}{4.44^2} \times J = 24.68\,J \quad (9)$$

Where X_P is a constant, a_e is the radius of the solute (TargetX), and r_L and r_S are radiuses of large and small pores, respectively. Peclet numbers for large (Pe_L) and small pores (Pe_S) are:

$$Pe_L = \frac{J_L(1-\sigma_L)}{PS_L} = \frac{0.422\,J \times 0.967}{0.376\,J} = 1.085 \quad (10)$$

$$Pe_S = \frac{J_S(1-\sigma_S)}{PS_S} = \frac{0.578\,J \times 0.4849}{24.68\,J} = 0.0113 \quad (11)$$

It can be derived that the diffusive extravasation clearance of TargetX is:

$$\begin{aligned} CL_{diff} &= Diff \cdot J \cdot \left(1 - \frac{C_i}{C_v}\right) = \left(PS_L \cdot \frac{Pe_L}{e^{Pe_L} - 1} + PS_S \cdot \frac{Pe_S}{e^{Pe_S} - 1}\right) \cdot \left(1 - \frac{C_i}{C_v}\right) \\ &= \left(0.376 \times \frac{1.085}{e^{1.085} - 1} + 24.68 \times \frac{0.0113}{e^{0.0113} - 1}\right) \cdot J \cdot \left(1 - \frac{C_i}{C_v}\right) \end{aligned} \quad (12)$$

Therefore, the diffusion coefficient of TargetX, $Diff$, is calculated to be 24.75.

The glomerular sieving coefficient (θ) of TargetX is:

$$\theta = e^{1 - \frac{8.7}{1 + e^{0.028 \times \left(-\frac{MW}{1000} + 72.3\right)}}} = 0.655 \quad (13)$$

Therefore, its renal clearance (CL_R) is:

$$CL_R = GFR \times \theta = 0.154 \times 0.655 = 0.1\ (L/h) \quad (14)$$

Where GFR is rat glomerular filtration rate.

Wu et al. estimated the permeability-surface area product of mAb (PS_{PV}^{mAb}) in perivascular space of brain to be 2.62×10^{-7} L/h [1]. To gain the value of $PS_{PV}^{TargetX}$, we utilized the following equation [2]:

$$\frac{PS_{PV}^{TargetX}}{PS_{PV}^{mAb}} = \frac{(-10.43 \times ln\frac{MW_{TargetX}}{1000} + 53.46) \times \frac{3600}{1000}}{(-10.43 \times ln\frac{MW_{mAb}}{1000} + 53.46) \times \frac{3600}{1000}} = 21.63 \tag{15}$$

Therefore, $PS_{PV}^{TargetX}$ was set to $5.667 \times 10^{-6}\ L/h$.

The volume of meningeal cells was estimated using the brain surface area and assuming a single cell layer of pia mater and arachnoid mater surrounding the CSF. According to the rat brain atlas by Paxinos, George, and Charles Watson (The Rat Brain in Stereotaxic Coordinates: Hard Cover Edition, available online via Elsevier, 2006), the brain surface area is approximately 12 cm^2. The total volume of the layer comes to 0.024 mL assuming that each cell layer is 10 μm thick.

QSP Model Equations

This section provides differential equations for the AAV-mAb-TargetX QSP model. Initial conditions of state variables are 0 unless otherwise specified.

AAV in the vascular space of an organ excluding brain:

$$V_{org}^{V}\frac{dC_{org}^{V,AAV}}{dt} = Mass_{org}^{VI,AAV} - Mass_{org}^{VO,AAV} - \left(1-\sigma_{org}^{V,AAV}\right)\cdot J_{org}\cdot C_{org}^{V,AAV} + \left(-K_{on}^{AAV}\cdot C_{org}^{V,AAV}\cdot R_{org}^{E,CML} + K_{off}^{AAV}\cdot RC_{org}^{E,CML}\right)\cdot V_{org}^{CV,E} + \left(-K_{on}^{AAV}\cdot C_{org}^{V,AAV}\cdot R_{org}^{BC,CM} + K_{off}^{AAV}\cdot RC_{org}^{BC,CM}\right)\cdot V_{org}^{BC} \quad (16)$$

Where masses of AAV entering and exiting lung are expressed as the following:

$$Mass_{Lung}^{VI,AAV} = (Q_{Lung} + J_{Lung})\cdot C_{Plasma}^{AAV} \quad (17)$$

$$Mass_{Lung}^{VO,AAV} = Q_{Lung}\cdot C_{Lung}^{V,AAV} \quad (18)$$

Whereas for liver,

$$Mass_{Liver}^{VI,AAV} = Q_{Liver}\cdot C_{Lung}^{V,AAV} + \sum_{i=Pancreas,SI,LI,spleen}(Q_i - J_i)\cdot C_i^{V,AAV} \quad (19)$$

$$Mass_{Liver}^{VO,AAV} = ((Q_{Liver} - J_{Liver}) + \sum_{i=Pancreas,SI,LI,spleen}(Q_i - J_i))\cdot C_{Liver}^{V,AAV} \quad (20)$$

For all other organs,

$$Mass_{org}^{VI,AAV} = Q_{org}\cdot C_{Lung}^{V,AAV} \quad (21)$$

$$Mass_{org}^{VO,AAV} = \left(Q_{org} - J_{org}\right)\cdot C_{org}^{V,AAV} \quad (22)$$

Free AAV receptor concentration on the luminal cell membrane of endothelial cells in an organ excluding brain:

$$\frac{dR_{org}^{E,CML}}{dt} = -K_{on}^{AAV} \cdot C_{org}^{V,AAV} \cdot R_{org}^{E,CML} + K_{off}^{AAV} \cdot RC_{org}^{E,CML} + K_{rec} \cdot R_{org}^{E,E} - K_{int} \cdot R_{org}^{E,CML}$$
$$R_{org}^{E,CML}(0) = R_{tot}^{org} \quad (23)$$

Bound AAV-receptor complex concentration on the luminal cell membrane of endothelial cells in an organ excluding brain:

$$\frac{dRC_{org}^{E,CML}}{dt} = K_{on}^{AAV} \cdot C_{org}^{V,AAV} \cdot R_{org}^{E,CML} - K_{off}^{AAV} \cdot RC_{org}^{E,CML} + K_{rec} \cdot RC_{org}^{E,E} - K_{int} \cdot RC_{org}^{E,CML} \quad (24)$$

Free AAV receptor concentration on the abluminal cell membrane of endothelial cells in an organ excluding brain:

$$\frac{dR_{org}^{E,CMAL}}{dt} = -K_{on}^{AAV} \cdot C_{org}^{I,AAV} \cdot R_{org}^{E,CMAL} + K_{off}^{AAV} \cdot RC_{org}^{E,CMAL} + K_{trans} \cdot R_{org}^{E,E} - K_{int} \cdot R_{org}^{E,CMAL}$$
$$R_{org}^{E,CMAL}(0) = R_{tot}^{org} \quad (25)$$

Bound AAV-receptor complex concentration on the abluminal cell membrane of endothelial cells in an organ excluding brain:

$$\frac{dRC_{org}^{E,CMAL}}{dt} = K_{on}^{AAV} \cdot C_{org}^{I,AAV} \cdot R_{org}^{E,CMAL} - K_{off}^{AAV} \cdot RC_{org}^{E,CMAL} + K_{trans} \cdot RC_{org}^{E,E} - K_{int} \cdot RC_{org}^{E,CMAL} \quad (26)$$

Free intracellular AAV receptor concentration in endothelial cells of an organ excluding brain:

$$\frac{dR_{org}^{E,E}}{dt} = -K_{on}^{AAV} \cdot C_{org}^{E,E,AAV} \cdot R_{org}^{E,E} + K_{off}^{AAV} \cdot RC_{org}^{E,E} - (K_{rec} + K_{trans}) \cdot R_{org}^{E,E} + K_{int} \cdot (R_{org}^{E,CML} + R_{org}^{E,CMAL}) \tag{27}$$

Free intracellular AAV concentration in endothelial cells of an organ excluding brain:

$$\frac{dC_{org}^{E,E,AAV}}{dt} = -K_{on}^{AAV} \cdot C_{org}^{E,E,AAV} \cdot R_{org}^{E,E} + K_{off}^{AAV} \cdot RC_{org}^{E,E} - K_{deg}^{AAV} \cdot C_{org}^{E,E,AAV} - K_{nuc}^{AAV} \cdot C_{org}^{E,E,AAV} \tag{28}$$

Bound intracellular AAV-receptor complex concentration in endothelial cells of an organ excluding brain:

$$\frac{dRC_{org}^{E,E}}{dt} = K_{on}^{AAV} \cdot C_{org}^{E,E,AAV} \cdot R_{org}^{E,E} - K_{off}^{AAV} \cdot RC_{org}^{E,E} - (K_{rec} + K_{trans}) \cdot RC_{org}^{E,E} + K_{int} \cdot (RC_{org}^{E,CML} + RC_{org}^{E,CMAL}) \tag{29}$$

Nuclear episomal, double-stranded AAV DNA concentration in endothelial cells of an organ excluding brain:

$$\frac{dC_{org}^{E,N,AAV}}{dt} = K_{nuc}^{AAV} \cdot C_{org}^{E,E,AAV} - K_{loss}^{org} \cdot C_{org}^{E,N,AAV} \tag{30}$$

AAV in the interstitial space excluding brain:

$$V_{org}^{I} \frac{dC_{org}^{I,AAV}}{dt} = \left(1 - \sigma_{org}^{V,AAV}\right) \cdot J_{org} \cdot C_{org}^{V,AAV} - \left(1 - \sigma_{org}^{I}\right) \cdot J_{org} \cdot C_{org}^{I,AAV} + \left(-K_{on}^{AAV} \cdot C_{org}^{I,AAV} \cdot R_{org}^{E,CMAL} + K_{off}^{AAV} \cdot RC_{org}^{E,CMAL}\right) \cdot V_{org}^{CV,E} + (-K_{on}^{AAV} \cdot C_{org}^{I,AAV} \cdot R_{org}^{P,CM} + K_{off}^{AAV} \cdot RC_{org}^{P,CM}) \cdot V_{org}^{CV,P} \tag{31}$$

Free AAV receptor concentration on parenchymal cell membrane in an organ:

$$\frac{dR_{org}^{P,CM}}{dt} = -K_{on}^{AAV} \cdot C_{org}^{I,AAV} \cdot R_{org}^{P,CM} + K_{off}^{AAV} \cdot RC_{org}^{P,CM} + K_{rec} \cdot R_{org}^{P,E} - K_{int} \cdot R_{org}^{P,CM}$$

$$R_{org}^{P,CM}(0) = R_{tot}^{org} \quad (32)$$

Bound AAV-receptor complex concentration on parenchymal cell membrane of endothelial cells in an organ:

$$\frac{dRC_{org}^{P,CM}}{dt} = K_{on}^{AAV} \cdot C_{org}^{I,AAV} \cdot R_{org}^{P,CM} - K_{off}^{AAV} \cdot RC_{org}^{P,CM} + K_{rec} \cdot RC_{org}^{P,E} - K_{int} \cdot RC_{org}^{P,CM} \quad (33)$$

Free intracellular AAV receptor concentration in parenchymal cells of an organ:

$$\frac{dR_{org}^{P,E}}{dt} = -K_{on}^{AAV} \cdot C_{org}^{P,E,AAV} \cdot R_{org}^{P,E} + K_{off}^{AAV} \cdot RC_{org}^{P,E} - K_{rec} \cdot R_{org}^{P,E} + K_{int} \cdot R_{org}^{P,CM} \quad (34)$$

Free intracellular AAV concentration in parenchymal cells of an organ:

$$\frac{dC_{org}^{P,E,AAV}}{dt} = -K_{on}^{AAV} \cdot C_{org}^{P,E,AAV} \cdot R_{org}^{P,E} + K_{off}^{AAV} \cdot RC_{org}^{P,E} - K_{deg}^{AAV} \cdot C_{org}^{P,E,AAV} - K_{nuc}^{AAV} \cdot C_{org}^{P,E,AAV} \quad (35)$$

Bound intracellular AAV-receptor complex concentration in parenchymal cells of an organ:

$$\frac{dRC_{org}^{P,E}}{dt} = K_{on}^{AAV} \cdot C_{org}^{P,E,AAV} \cdot R_{org}^{P,E} - K_{off}^{AAV} \cdot RC_{org}^{P,E} - K_{rec} \cdot RC_{org}^{P,E} + K_{int} \cdot RC_{org}^{P,E} \quad (36)$$

Nuclear episomal, double-stranded AAV DNA concentration in parenchymal cells of an organ:

$$\frac{dC_{org}^{P,N,AAV}}{dt} = K_{nuc}^{AAV} \cdot C_{org}^{P,E,AAV} - K_{loss}^{org} \cdot C_{org}^{P,N,AAV} \tag{37}$$

Free AAV receptor concentration on blood cell membrane in an organ:

$$V_{org}^{BC} \frac{dR_{org}^{BC,CM}}{dt} = \left(-K_{on}^{AAV} \cdot C_{org}^{V,AAV} \cdot R_{org}^{BC,CM} + K_{off}^{AAV} \cdot RC_{org}^{BC,CM} + K_{rec} \cdot R_{org}^{BC,E} - K_{int} \cdot R_{org}^{BC,CM}\right) \cdot V_{org}^{BC} + MT\_R_{org}^{BC,CM} \tag{38}$$

$$R_{org}^{BC,CM}(0) = R_{tot}^{BC}$$

Bound AAV-receptor complex concentration on blood cell membrane in an organ:

$$V_{org}^{BC} \frac{dRC_{org}^{BC,CM}}{dt} = \left(K_{on}^{AAV} \cdot C_{org}^{V,AAV} \cdot R_{org}^{BC,CM} - K_{off}^{AAV} \cdot RC_{org}^{BC,CM} + K_{rec} \cdot RC_{org}^{BC,E} - K_{int} \cdot RC_{org}^{BC,CM}\right) \cdot V_{org}^{BC} + MT\_RC_{org}^{BC,CM} \tag{39}$$

Free intracellular AAV receptor concentration in blood cells of an organ:

$$V_{org}^{BC} \frac{dR_{org}^{BC,E}}{dt} = \left(-K_{on}^{AAV} \cdot C_{org}^{BC,E,AAV} \cdot R_{org}^{BC,E} + K_{off}^{AAV} \cdot RC_{org}^{BC,E} - K_{rec} \cdot R_{org}^{BC,E} + K_{int} \cdot R_{org}^{BC,CM}\right) \cdot V_{org}^{BC} + MT\_R_{org}^{BC,E} \tag{40}$$

Free intracellular AAV concentration in blood cells of an organ:

$$V_{org}^{BC} \frac{dC_{org}^{BC,E,AAV}}{dt} = \left(-K_{on}^{AAV} \cdot C_{org}^{BC,E,AAV} \cdot R_{org}^{BC,E} + K_{off}^{AAV} \cdot RC_{org}^{BC,E} - K_{deg}^{AAV} \cdot C_{org}^{BC,E,AAV} - K_{nuc}^{AAV} \cdot C_{org}^{BC,E,AAV}\right) \cdot V_{org}^{BC} + MT\_C_{org}^{BC,E,AAV} \tag{41}$$

Bound intracellular AAV-receptor complex concentration in blood cells of an organ:

$$V_{org}^{BC}\frac{dRC_{org}^{BC,E}}{dt}=\left(K_{on}^{AAV}\cdot C_{org}^{BC,E,AAV}\cdot R_{org}^{BC,E}-K_{off}^{AAV}\cdot RC_{org}^{BC,E}+K_{int}\cdot RC_{org}^{BC,CM}-K_{rec}\cdot RC_{org}^{BC,E}\right)\cdot V_{org}^{BC}+MT\_RC_{org}^{BC,E} \quad (42)$$

"Nuclear" episomal, double-stranded AAV DNA concentration in blood cells of an organ:

$$V_{org}^{BC}\frac{dC_{org}^{BC,N,AAV}}{dt}=(K_{nuc}^{AAV}\cdot C_{org}^{BC,E,AAV}-K_{loss}^{BC}\cdot C_{org}^{BC,N,AAV})\cdot V_{org}^{BC}+MT\_C_{org}^{BC,N,AAV} \quad (43)$$

Since blood cells circulate throughout the body, mass transfers for all the blood cell-associated species in organs excluding lung and liver are expressed as:

$$MT\_X_{org}=BCQ_{org}\cdot(X_{Lung}-X_{org}) \quad (44)$$

Where X_{org} represents $R_{org}^{BC,CM}$, $RC_{org}^{BC,CM}$, $R_{org}^{BC,E}$, $C_{org}^{BC,E,AAV}$, $RC_{org}^{BC,E}$, or $C_{org}^{BC,N,AAV}$.

In particular, for lung and liver,

$$MT\_X_{Lung}=BCQ_{Lung}\cdot(X_{BC,central}-X_{Lung}) \quad (45)$$

$$MT\_X_{Liver}=BCQ_{Liver}\cdot\left(X_{Lung}-X_{Liver}\right)+\sum_{i=Pancreas,SI,LI,spleen}BCQ_i\cdot(X_i-X_{Liver}) \quad (46)$$

Because of the more complex anatomical representation of brain in the model, equations specific to brain sub-regions are described below.

AAV in the vascular space of brain:

$$V_{Brain}^{V}\frac{dC_{Brain}^{V,AAV}}{dt} = Mass_{Brain}^{VI,AAV} - Mass_{Brain}^{VO,AAV} - \left(1-\sigma_{BBB}^{V,AAV}\right)\cdot Q_{ISF}\cdot C_{Brain}^{V,AAV} - \left(1-\sigma_{BCSFB}^{V,AAV}\right)\cdot Q_{CSF}\cdot C_{Brain}^{V,AAV} + \left(-K_{on}^{AAV}\cdot C_{Brain}^{V,AAV}\cdot R_{BBB}^{E,CML} + K_{off}^{AAV}\cdot RC_{BBB}^{E,CML}\right)\cdot\left(V_{Brain}^{CV,E}\cdot\frac{SA_{BBB}}{SA_{BBB}+SA_{BCSFB}}\right) + \left(-K_{on}^{AAV}\cdot C_{Brain}^{V,AAV}\cdot R_{BCSFB}^{E,CML} + K_{off}^{AAV}\cdot RC_{BCSFB}^{E,CML}\right)\cdot\left(V_{Brain}^{CV,E}\cdot\frac{SA_{BCSFB}}{SA_{BBB}+SA_{BCSFB}}\right) + \left(-K_{on}^{AAV}\cdot C_{Brain}^{V,AAV}\cdot R_{Brain}^{BC,CM} + K_{off}^{AAV}\cdot RC_{Brain}^{BC,CM}\right)\cdot V_{Brain}^{BC} \tag{47}$$

Free AAV receptor concentration on the luminal cell membrane of endothelial cells in brain:

$$\frac{dR_{Barrier}^{E,CML}}{dt} = -K_{on}^{AAV}\cdot C_{Brain}^{V,AAV}\cdot R_{Barrier}^{E,CML} + K_{off}^{AAV}\cdot RC_{Barrier}^{E,CML} + K_{rec}\cdot R_{Barrier}^{E,E} - K_{int}\cdot R_{Barrier}^{E,CML} \tag{48}$$

$$R_{Barrier}^{E,CML}(0) = R_{tot}^{Brain}$$

Where the subscript "$Barrier$" refers to either "BBB" or "$BCSFB$".

Bound AAV-receptor complex concentration on the luminal cell membrane of endothelial cells in brain:

$$\frac{dRC_{Barrier}^{E,CML}}{dt} = K_{on}^{AAV}\cdot C_{Brain}^{V,AAV}\cdot R_{Barrier}^{E,CML} - K_{off}^{AAV}\cdot RC_{Barrier}^{E,CML} + K_{rec}\cdot RC_{Barrier}^{E,E} - K_{int}\cdot RC_{Barrier}^{E,CML} \tag{49}$$

Free intracellular AAV receptor concentration in endothelial cells of brain:

$$\frac{dR_{Barrier}^{E,E}}{dt} = -K_{on}^{AAV} \cdot C_{Barrier}^{E,E,AAV} \cdot R_{Barrier}^{E,E} + K_{off}^{AAV} \cdot RC_{Barrier}^{E,E} - (K_{rec} + K_{trans}) \cdot R_{Barrier}^{E,E} + K_{int} \cdot (R_{Barrier}^{E,CML} + R_{Barrier}^{E,CMAL}) \tag{50}$$

Free intracellular AAV concentration in endothelial cells of brain:

$$\frac{dC_{Barrier}^{E,E,AAV}}{dt} = -K_{on}^{AAV} \cdot C_{Barrier}^{E,E,AAV} \cdot R_{Barrier}^{E,E} + K_{off}^{AAV} \cdot RC_{Barrier}^{E,E} - K_{deg}^{AAV} \cdot C_{Barrier}^{E,E,AAV} - K_{nuc}^{AAV} \cdot C_{Barrier}^{E,E,AAV} \tag{51}$$

Bound intracellular AAV-receptor complex concentration in endothelial cells of brain:

$$\frac{dRC_{Barrier}^{E,E}}{dt} = K_{on}^{AAV} \cdot C_{Barrier}^{E,E,AAV} \cdot R_{Barrier}^{E,E} - K_{off}^{AAV} \cdot RC_{Barrier}^{E,E} - (K_{rec} + K_{trans}) \cdot RC_{Barrier}^{E,E} + K_{int} \cdot (RC_{Barrier}^{E,CML} + RC_{Barrier}^{E,CMAL}) \tag{52}$$

Free AAV receptor concentration on the abluminal cell membrane of endothelial BBB cells in brain:

$$\frac{dR_{BBB}^{E,CMAL}}{dt} = -K_{on}^{AAV} \cdot C_{Brain}^{I,AAV} \cdot R_{org}^{E,CMAL} + K_{off}^{AAV} \cdot RC_{BBB}^{E,CMAL} + K_{trans} \cdot R_{BBB}^{E,E} - K_{int} \cdot R_{BBB}^{E,CMAL} \tag{53}$$

$$R_{org}^{E,CMAL}(0) = R_{tot}^{Brain}$$

Bound AAV-receptor complex concentration on the abluminal cell membrane of endothelial BBB cells in brain:

$$\frac{dRC_{BBB}^{E,CMAL}}{dt} = K_{on}^{AAV} \cdot C_{Brain}^{I,AAV} \cdot R_{BBB}^{E,CMAL} - K_{off}^{AAV} \cdot RC_{BBB}^{E,CMAL} + K_{trans} \cdot RC_{BBB}^{E,E} - K_{int} \cdot RC_{BBB}^{E,CMAL} \tag{54}$$

Free AAV receptor concentration on the abluminal cell membrane of endothelial BCSFB cells in brain:

$$\begin{aligned}\frac{dR_{BCSFB}^{E,CMAL}}{dt} &= \left(-K_{on}^{AAV} \cdot C_{Brain}^{LV,AAV} \cdot R_{BCSFB}^{E,CMAL} + K_{off}^{AAV} \cdot RC_{BCSFB}^{E,CMAL}\right) \cdot \frac{V_{Brain}^{LV}}{V_{Brain}^{LV} + V_{Brain}^{TFV}} \\ &+ \left(-K_{on}^{AAV} \cdot C_{Brain}^{TFV,AAV} \cdot R_{BCSFB}^{E,CMAL} + K_{off}^{AAV} \cdot RC_{BCSFB}^{E,CMAL}\right) \cdot \frac{V_{Brain}^{TFV}}{V_{Brain}^{LV} + V_{Brain}^{TFV}} \\ &+ K_{trans} \cdot R_{BCSFB}^{E,E} - K_{int} \cdot R_{BCSFB}^{E,CMAL}\end{aligned} \tag{55}$$

$$R_{BCSFB}^{E,CMAL}(0) = R_{tot}^{Brain}$$

Bound AAV-receptor complex concentration on the abluminal cell membrane of endothelial BCSFB cells in brain:

$$\begin{aligned}\frac{dRC_{BCSFB}^{E,CMAL}}{dt} &= \left(K_{on}^{AAV} \cdot C_{Brain}^{LV,AAV} \cdot R_{BCSFB}^{E,CMAL} - K_{off}^{AAV} \cdot RC_{BCSFB}^{E,CMAL}\right) \cdot \frac{V_{Brain}^{LV}}{V_{Brain}^{LV} + V_{Brain}^{TFV}} \\ &+ \left(K_{on}^{AAV} \cdot C_{Brain}^{TFV,AAV} \cdot R_{BCSFB}^{E,CMAL} - K_{off}^{AAV} \cdot RC_{BCSFB}^{E,CMAL}\right) \cdot \frac{V_{Brain}^{TFV}}{V_{Brain}^{LV} + V_{Brain}^{TFV}} \\ &+ K_{trans} \cdot RC_{BCSFB}^{E,E} - K_{int} \cdot RC_{BCSFB}^{E,CMAL}\end{aligned} \tag{56}$$

Nuclear episomal, double-stranded AAV DNA concentration in brain endothelial cells:

$$\frac{dC_{Barrier}^{E,N,AAV}}{dt} = K_{nuc}^{AAV} \cdot C_{Barrier}^{E,E,AAV} - K_{loss}^{Brain} \cdot C_{Barrier}^{E,N,AAV} \tag{57}$$

AAV in LV:

$$V_{Brain}^{LV}\frac{dC_{Brain}^{LV,AAV}}{dt} = \left(1-\sigma_{BCSFB}^{V,AAV}\right)\cdot\left(Q_{CSF}\cdot\frac{V_{Brain}^{LV}}{V_{Brain}^{LV}+V_{Brain}^{TFV}}\right)\cdot C_{Brain}^{V,AAV} - \left(1-\sigma_{CSF}^{LV-TFV,AAV}\right)\cdot\left(Q_{CSF}\cdot\frac{V_{Brain}^{LV}}{V_{Brain}^{LV}+V_{Brain}^{TFV}}\right)\cdot C_{Brain}^{LV,AAV} + \left(-K_{on}^{AAV}\cdot C_{Brain}^{LV,AAV}\cdot R_{BCSFB}^{E,CMAL}+K_{off}^{AAV}\cdot RC_{BCSFB}^{E,CMAL}\right)\cdot\frac{V_{Brain}^{LV}}{V_{Brain}^{LV}+V_{Brain}^{TFV}}\cdot\left(V_{Brain}^{CV,E}\cdot\frac{SA_{BCSFB}}{SA_{BBB}+SA_{BCSFB}}\right) \tag{58}$$

AAV in TFV:

$$V_{Brain}^{TFV}\frac{dC_{Brain}^{TFV,AAV}}{dt} = \left(1-\sigma_{BCSFB}^{V,AAV}\right)\cdot\left(Q_{CSF}\cdot\frac{V_{Brain}^{TFV}}{V_{Brain}^{LV}+V_{Brain}^{TFV}}\right)\cdot C_{Brain}^{V,AAV} + \left(1-\sigma_{CSF}^{LV-TFV,AAV}\right)\cdot\left(Q_{CSF}\cdot\frac{V_{Brain}^{LV}}{V_{Brain}^{LV}+V_{Brain}^{TFV}}\right)\cdot C_{Brain}^{LV,AAV} - \left(1-\sigma_{CSF}^{TFV-CM,AAV}\right)\cdot Q_{CSF}\cdot C_{Brain}^{TFV,AAV} + \left(-K_{on}^{AAV}\cdot C_{Brain}^{TFV,AAV}\cdot R_{BCSFB}^{E,CMAL}+K_{off}^{AAV}\cdot RC_{BCSFB}^{E,CMAL}\right)\cdot\frac{V_{Brain}^{TFV}}{V_{Brain}^{LV}+V_{Brain}^{TFV}}\cdot\left(V_{Brain}^{CV,E}\cdot\frac{SA_{BCSFB}}{SA_{BBB}+SA_{BCSFB}}\right) \tag{59}$$

Free AAV in CM:

$$V_{Brain}^{CM}\frac{dC_{Brain}^{CM,AAV}}{dt}$$

$$= Infusion_{CM} + \left(1-\sigma_{CSF}^{TFV-CM,AAV}\right)\cdot Q_{CSF}\cdot C_{Brain}^{TFV,AAV} + Q_{CSF}^{OSC}\cdot C_{Brain}^{SAS,AAV}$$
$$- \left(1-\sigma_{CSF}^{CM-SAS,AAV}\right)\cdot\left(Q_{CSF}+Q_{CSF}^{OSC}\right)\cdot C_{Brain}^{CM,AAV} + (-K_{on}^{AAV}\cdot C_{Brain}^{CM,AAV}\cdot R_{CM}^{MEN,CM} \quad (60)$$
$$+ K_{off}^{AAV}\cdot RC_{CM}^{MEN,CM})\cdot(V_{Brain}^{MEN}\cdot\frac{V_{Brain}^{CM}}{V_{Brain}^{CM}+V_{Brain}^{SAS}})$$

Where "$Infusion_{CM}$" is used to characterize data from intra-cisternal magna administration.

Free AAV in SAS:

$$V_{Brain}^{SAS}\frac{dC_{Brain}^{SAS,AAV}}{dt}$$

$$= \left(1-\sigma_{CSF}^{CM-SAS,AAV}\right)\cdot\left(Q_{CSF}+Q_{CSF}^{OSC}\right)\cdot C_{Brain}^{CM,AAV} - (1-\sigma_{SAS}^{I})\cdot Q_{CSF}\cdot C_{Brain}^{SAS,AAV}$$
$$- Q_{CSF}^{OSC}\cdot C_{Brain}^{SAS,AAV} - Q_{PV}\cdot C_{Brain}^{SAS,AAV} + (Q_{ISF}\cdot(1-FR_{lymph}^{AAV}) + Q_{PV})\cdot C_{Brain}^{PV,Ven,AAV} \quad (61)$$
$$+ (-K_{on}^{AAV}\cdot C_{Brain}^{SAS,AAV}\cdot R_{SAS}^{MEN,CM} + K_{off}^{AAV}\cdot RC_{SAS}^{MEN,CM})\cdot(V_{Brain}^{MEN}\cdot\frac{V_{Brain}^{SAS}}{V_{Brain}^{CM}+V_{Brain}^{SAS}})$$

Free AAV receptor concentration on cell membrane of meningeal cells:

$$\frac{dR_{Z}^{MEN,CM}}{dt} = -K_{on}^{AAV}\cdot C_{Brain}^{Z,AAV}\cdot R_{Z}^{MEN,CM} + K_{off}^{AAV}\cdot RC_{Z}^{MEN,CM} + K_{rec}\cdot R_{Z}^{MEN,E} - K_{int}$$
$$\cdot R_{Z}^{MEN,CM} \quad (62)$$

$$R_{Z}^{MEN,CM}(0) = R_{tot}^{MEN}$$

Where the subscript "Z" refers to either "CM" or "SAS".

Bound AAV-receptor complex concentration on the cell membrane of meningeal cells:

$$\frac{dRC_Z^{MEN,CM}}{dt} = K_{on}^{AAV} \cdot C_{Brain}^{Z,AAV} \cdot R_Z^{MEN,CM} - K_{off}^{AAV} \cdot RC_Z^{MEN,CM} + K_{rec} \cdot RC_Z^{MEN,E} - K_{int} \cdot RC_Z^{MEN,CM} \tag{63}$$

Free intracellular AAV receptor concentration in meningeal cells:

$$\frac{dR_Z^{MEN,E}}{dt} = -K_{on}^{AAV} \cdot C_Z^{MEN,E,AAV} \cdot R_Z^{MEN,E} + K_{off}^{AAV} \cdot RC_Z^{MEN,E} - K_{rec} \cdot R_Z^{MEN,E} + K_{int} \cdot R_Z^{MEN,CM} \tag{64}$$

Free intracellular AAV concentration in meningeal cells:

$$\frac{dC_Z^{MEN,E,AAV}}{dt} = -K_{on}^{AAV} \cdot C_Z^{MEN,E,AAV} \cdot R_Z^{MEN,E} + K_{off}^{AAV} \cdot RC_Z^{MEN,E} - K_{deg}^{AAV} \cdot C_Z^{MEN,E,AAV} - K_{nuc}^{AAV} \cdot C_Z^{MEN,E,AAV} \tag{65}$$

Bound intracellular AAV-receptor complex concentration in meningeal cells:

$$\frac{dRC_Z^{MEN,E}}{dt} = K_{on}^{AAV} \cdot C_Z^{MEN,E,AAV} \cdot R_Z^{MEN,E} - K_{off}^{AAV} \cdot RC_Z^{MEN,E} - K_{rec} \cdot RC_Z^{MEN,E} + K_{int} \cdot RC_Z^{MEN,CM} \tag{66}$$

Nuclear episomal, double-stranded AAV DNA concentration in meningeal cells:

$$\frac{dC_Z^{MEN,N,AAV}}{dt} = K_{nuc}^{AAV} \cdot C_Z^{MEN,E,AAV} - K_{loss}^{Brain} \cdot C_Z^{MEN,N,AAV} \tag{67}$$

AAV in the interstitial space of brain:

$$V_{Brain}^{I} \frac{dC_{Brain}^{I,AAV}}{dt} = \left(1 - \sigma_{BBB}^{V,AAV}\right) \cdot Q_{ISF} \cdot C_{Brain}^{V,AAV} - \left(1 - \sigma_{Brain}^{I}\right) \cdot Q_{ISF} \cdot C_{Brain}^{I,AAV} + PS_{PV}^{AAV} \cdot \left(C_{Brain}^{PV,Art,AAV} - C_{Brain}^{I,AAV}\right) + PS_{PV}^{AAV} \cdot \left(C_{Brain}^{PV,Ven,AAV} - C_{Brain}^{I,AAV}\right) + \left(-K_{on}^{AAV} \cdot C_{Brain}^{I,AAV} \cdot R_{Brain}^{P,CM} + K_{off}^{AAV} \cdot RC_{Brain}^{P,CM}\right) \cdot V_{Brain}^{CV,P} + \left(-K_{on}^{AAV} \cdot C_{org}^{I,AAV} \cdot R_{BBB}^{E,CMAL} + K_{off}^{AAV} \cdot RC_{BBB}^{E,CMAL}\right) \cdot V_{Brain}^{CV,E} \cdot \frac{SA_{BBB}}{SA_{BBB} + SA_{BCSFB}} \quad (68)$$

AAV in perivascular arterial space:

$$V_{Brain}^{PV,Art} \frac{dC_{Brain}^{PV,Art,AAV}}{dt} = Q_{PV} \cdot C_{Brain}^{SAS,AAV} - PS_{PV}^{AAV} \cdot \left(C_{Brain}^{PV,Art,AAV} - C_{Brain}^{I,AAV}\right) \quad (69)$$

$$V_{Brain}^{PV,Ven} \frac{dC_{Brain}^{PV,Ven,AAV}}{dt} = \left(1 - \sigma_{Brain}^{I}\right) \cdot Q_{ISF} \cdot C_{Brain}^{I,AAV} - (Q_{PV} + Q_{ISF}) \cdot C_{Brain}^{PV,Ven,AAV} - PS_{PV}^{AAV} \cdot \left(C_{Brain}^{PV,Ven,AAV} - C_{Brain}^{I,AAV}\right) \quad (70)$$

Ultimately, in central plasma and lymph:

$$V_{Plasma}\frac{dC_{Plasma}^{AAV}}{dt} = \sum_{\substack{i=All\ organs\ excluding\ brain,lung,\\ liver,SI,LI,pancreas,spleen}} (Q_i - J_i)\cdot C_i^{V,AAV} + (Q_{Brain} - Q_{ISF} - Q_{CSF})\cdot C_{Brain}^{V,AAV} + \left((Q_{Liver} - J_{Liver}) + \sum_{i=Pancreas,SI,LI,spleen}(Q_i - J_i)\right)\cdot C_{Liver}^{V,AAV} + L_{Lymph}\cdot C_{Lymph}^{AAV} - \left(Q_{Lung} + J_{Lung}\right)\cdot C_{Plasma}^{AAV} + (-K_{on}^{AAV}\cdot C_{Plasma}^{AAV}\cdot R_{Central}^{BC,CM} + K_{off}^{AAV}\cdot RC_{Central}^{BC,CM})\cdot V_{BC} \tag{71}$$

$$V_{Lymph}\frac{dC_{Lymph}^{AAV}}{dt} = \sum_{i=All\ organs\ excluding\ brain}\left(1-\sigma_i^I\right)\cdot J_i\cdot C_i^{I,AAV} + (Q_{ISF}\cdot FR_{lymph}^{AAV})\cdot C_{Brain}^{PV,Ven,AAV} + (1-\sigma_{SAS}^I)\cdot Q_{CSF}\cdot C_{Brain}^{SAS,AAV} - L_{Lymph}\cdot C_{Lymph}^{AAV} \tag{72}$$

In a similar fashion to equations describing blood cells in each organ, free AAV receptor concentration on cell membrane, bound AAV-receptor complex concentration on cell membrane, intracellular free AAV receptor, intracellular free AAV, intracellular bound AAV-receptor complex, and nuclear vector of central blood cells are expressed as equations (58)-(63).

$$V_{BC}\frac{dR_{Central}^{BC,CM}}{dt} = \left(-K_{on}^{AAV} \cdot C_{Plasma}^{AAV} \cdot R_{Central}^{BC,CM} + K_{off}^{AAV} \cdot RC_{Central}^{BC,CM} + K_{rec} \cdot R_{Central}^{BC,E} - K_{int} \cdot R_{Central}^{BC,CM}\right) \cdot V_{BC} - BCQ_{Lung} \cdot R_{Central}^{BC,CM} + \sum_{\substack{i=All\ organs\ excluding\ lung,\\ liver,SI,LI,pancreas,spleen}} BCQ_i \cdot R_i^{BC,CM} + \sum_{i=Liver,SI,LI,pancreas,spleen} BCQ_i \cdot R_{Liver}^{BC,CM} \tag{73}$$

$$R_{Central}^{BC,CM}(0) = R_{tot}^{BC}$$

$$V_{BC}\frac{dRC_{Central}^{BC,CM}}{dt} = \left(K_{on}^{AAV} \cdot C_{Plasma}^{AAV} \cdot R_{Central}^{BC,CM} - K_{off}^{AAV} \cdot RC_{Central}^{BC,CM} + K_{rec} \cdot RC_{Central}^{BC,E} - K_{int} \cdot RC_{Central}^{BC,CM}\right) \cdot V_{BC} - BCQ_{Lung} \cdot RC_{Central}^{BC,CM} + \sum_{\substack{i=All\ organs\ excluding\ lung,\\ liver,SI,LI,pancreas,spleen}} BCQ_i \cdot RC_i^{BC,CM} + \sum_{i=Liver,SI,LI,pancreas,spleen} BCQ_i \cdot RC_{Liver}^{BC,CM} \tag{74}$$

$$V_{BC}\frac{dR_{Central}^{BC,E}}{dt} = \left(-K_{on}^{AAV} \cdot C_{Central}^{BC,E,AAV} \cdot R_{Central}^{BC,E} + K_{off}^{AAV} \cdot RC_{Central}^{BC,E} - K_{rec} \cdot R_{Central}^{BC,E} + K_{int} \cdot R_{Central}^{BC,CM}\right) \cdot V_{BC} - BCQ_{Lung} \cdot R_{Central}^{BC,E} + \sum_{\substack{i=All\ organs\ excluding\ lung,\\ liver,SI,LI,pancreas,spleen}} BCQ_i \cdot R_i^{BC,E} + \sum_{i=Liver,SI,LI,pancreas,spleen} BCQ_i \cdot R_{Liver}^{BC,E} \tag{75}$$

$$V_{BC}\frac{dC_{Central}^{BC,E,AAV}}{dt} = \left(-K_{on}^{AAV}\cdot C_{Central}^{BC,E,AAV}\cdot R_{Central}^{BC,E} + K_{off}^{AAV}\cdot RC_{Central}^{BC,E} - K_{deg}^{AAV}\cdot C_{Central}^{BC,E,AAV} - K_{nuc}^{AAV}\cdot C_{Central}^{BC,E,AAV}\right)\cdot V_{BC} - BCQ_{Lung}\cdot C_{Central}^{BC,E,AAV} + \sum_{\substack{i=All\ organs\ excluding\ lung,\\ liver,SI,LI,pancreas,spleen}} BCQ_i\cdot C_i^{BC,E,AAV} + \sum_{i=Liver,SI,LI,pancreas,spleen} BCQ_i\cdot C_{Liver}^{BC,E,AAV} \tag{76}$$

$$V_{BC}\frac{dRC_{Central}^{BC,E}}{dt} = \left(K_{on}^{AAV}\cdot C_{Central}^{BC,E,AAV}\cdot R_{Central}^{BC,E} - K_{off}^{AAV}\cdot RC_{Central}^{BC,E} + K_{int}\cdot RC_{Central}^{BC,CM} - K_{rec}\cdot RC_{Central}^{BC,E}\right)\cdot V_{BC} - BCQ_{Lung}\cdot RC_{Central}^{BC,E} + \sum_{\substack{i=All\ organs\ excluding\ lung,\\ liver,SI,LI,pancreas,spleen}} BCQ_i\cdot RC_i^{BC,E} + \sum_{i=Liver,SI,LI,pancreas,spleen} BCQ_i\cdot RC_{Liver}^{BC,E} \tag{77}$$

$$V_{BC}\frac{dC_{Central}^{BC,N,AAV}}{dt} = (K_{nuc}^{AAV}\cdot C_{Central}^{BC,E,AAV} - K_{loss}^{BC}\cdot C_{Central}^{BC,N,AAV})\cdot V_{BC} \tag{78}$$

In the output, blood AAV in Vg/μL is expressed as

$$C_{Blood}^{AAV} = 10^{-6}\times(C_{Plasma}^{AAV}\cdot V_{Plasma} + (RC_{Central}^{BC,CM} + C_{Central}^{BC,E,AAV} + RC_{Central}^{BC,E} + 2\times C_{Central}^{BC,N,AAV})\cdot V_{BC})/(V_{Plasma}+V_{BC}) \tag{79}$$

A factor of 2 multiplied by nuclear vector concentrations indicate double-stranded forms of episomal DNA.

Tissue AAV concentrations in Vg/ng genomic DNA after perfusion is expressed as follows, assuming a cell volume of 3 pL/cell (literature suggests 1-10 pL) [3] and rat diploid genomic DNA of 6 pg/cell [4].

$$C_{org}^{AAV} = ((RC_{org}^{E,CML} + RC_{org}^{E,CMAL} + C_{org}^{E,E,AAV} + RC_{org}^{E,E} + 2 \times C_{org}^{E,N,AAV}) \cdot V_{org}^{CV,E} + C_{org}^{I,AAV} \cdot V_{org}^{I} + (RC_{org}^{P,CM} + C_{org}^{P,E,AAV} + RC_{org}^{P,E} + 2 \times C_{org}^{P,N,AAV}) \cdot V_{org}^{CV,P})/(6 \times 10^{-3}(V_{org}^{V} + V_{org}^{BC} + V_{org}^{I} + V_{org}^{C} + V_{org}^{E})/(3 \times 10^{-12})) \quad (80)$$

$$C_{Brain}^{AAV} = ((RC_{Brain}^{BBB,CML} + RC_{Brain}^{BBB,CMAL} + C_{Brain}^{BBB,E,AAV} + RC_{Brain}^{BBB,E} + 2 \times C_{Brain}^{BBB,N,AAV}) \cdot V_{Brain}^{CV,E} \cdot \frac{SA_{BBB}}{SA_{BBB} + SA_{BCSFB}} + \left(RC_{Brain}^{BCSFB,CML} + RC_{Brain}^{BCSFB,CMAL} + C_{Brain}^{BCSFB,E,AAV} + RC_{Brain}^{BCSFB,E} + 2 \times C_{Brain}^{BCSFB,N,AAV}\right) \cdot V_{Brain}^{CV,E} \cdot \frac{SA_{BCSFB}}{SA_{BBB} + SA_{BCSFB}} + C_{Brain}^{I,AAV} \cdot V_{Brain}^{I} + C_{Brain}^{LV,AAV} \cdot V_{Brain}^{LV} + C_{Brain}^{TFV,AAV} \cdot V_{Brain}^{TFV} + C_{Brain}^{PV,Art,AAV} \cdot V_{Brain}^{PV,Art} + C_{Brain}^{PV,Ven,AAV} \cdot V_{Brain}^{PV,Ven} + \left(RC_{CM}^{MEM,CM} + C_{CM}^{MEN,E,AAV} + RC_{CM}^{MEN,E} + 2 \times C_{CM}^{MEN,N,AAV}\right) \cdot V_{Brain}^{MEN} \cdot \frac{V_{Brain}^{CM}}{V_{Brain}^{CM} + V_{Brain}^{SAS}} + \left(RC_{SAS}^{MEM,CM} + C_{SAS}^{MEN,E,AAV} + RC_{SAS}^{MEN,E} + 2 \times C_{SAS}^{MEN,N,AAV}\right) \cdot V_{Brain}^{MEN} \cdot \frac{V_{Brain}^{SAS}}{V_{Brain}^{CM} + V_{Brain}^{SAS}} + (RC_{Brain}^{P,CM} + C_{Brain}^{P,E,AAV} + RC_{Brain}^{P,E} + 2 \times C_{Brain}^{P,N,AAV}) \cdot V_{Brain}^{CV,P})/(6 \times 10^{-3}(V_{Brain}^{V} + V_{Brain}^{BC} + V_{Brain}^{I} + V_{Brain}^{C} + V_{Brain}^{E} + V_{Brain}^{LV} + V_{Brain}^{TFV} + V_{Brain}^{PV})/(3 \times 10^{-12})) \quad (81)$$

Transgene expression connects the mAb PBPK model to the AAV PBPK model via a series of transit compartments.

Transit compartments in organ parenchymal cells:

$$\frac{dP1_{org}}{dt} = K_{prod}^{org} \cdot C_{org}^{P,N,AAV} - \frac{1}{\tau_{org}} \cdot P1_{org} \quad (82)$$

$$\frac{dP2_{org}}{dt} = \frac{1}{\tau_{org}} \cdot P1_{org} - \frac{1}{\tau_{org}} \cdot P2_{org} \quad (83)$$

$$\frac{dP3_{org}}{dt} = \frac{1}{\tau_{org}} \cdot P2_{org} - \frac{1}{\tau_{org}} \cdot P3_{org} \quad (84)$$

Produced intracellular mAb concentrations in organ parenchymal cells:

$$\frac{dC_{org}^{P,mAb}}{dt} = \frac{1}{\tau_{org}} \cdot P3_{org} - K_{sec}^{org} \cdot C_{org}^{P,mAb} \quad (85)$$

Whereas

$$C_{org}^{P,TargetX} = C_{org}^{P,comp} = 0 \quad (86)$$

Transit compartments in endothelial cells of organs other than brain:

$$\frac{dE1_{org}}{dt} = K_{prod}^{org} \cdot C_{org}^{E,N,AAV} - \frac{1}{\tau_{org}} \cdot E1_{org} \quad (87)$$

$$\frac{dE2_{org}}{dt} = \frac{1}{\tau_{org}} \cdot E1_{org} - \frac{1}{\tau_{org}} \cdot E2_{org} \quad (88)$$

$$\frac{dE3_{org}}{dt} = \frac{1}{\tau_{org}} \cdot E2_{org} - \frac{1}{\tau_{org}} \cdot E3_{org} \quad (89)$$

Produced intracellular mAb concentrations in endothelial cells of organs other than brain:

$$\frac{dC_{org}^{E,mAb}}{dt} = \frac{1}{\tau_{org}} \cdot E3_{org} - K_{sec}^{org} \cdot C_{org}^{E,mAb} \tag{90}$$

Whereas

$$C_{org}^{E,TargetX} = C_{org}^{E,comp} = 0 \tag{91}$$

Transit compartments in brain endothelial cells:

$$\frac{dE1_{Barrier}}{dt} = K_{prod}^{Brain} \cdot C_{Brain}^{E,N,AAV} - \frac{1}{\tau_{Brain}} \cdot E1_{Barrier} \tag{92}$$

$$\frac{dE2_{Barrier}}{dt} = \frac{1}{\tau_{Brain}} \cdot E1_{Barrier} - \frac{1}{\tau_{Brain}} \cdot E2_{Barrier} \tag{93}$$

$$\frac{dE3_{Barrier}}{dt} = \frac{1}{\tau_{Brain}} \cdot E2_{Barrier} - \frac{1}{\tau_{Brain}} \cdot E3_{Barrier} \tag{94}$$

Produced intracellular mAb concentrations in brain endothelial cells:

$$\frac{dC_{Barrier}^{E,mAb}}{dt} = \frac{1}{\tau_{org}} \cdot E3_{Barrier} - K_{sec}^{Brain} \cdot C_{Barrier}^{E,mAb} \tag{95}$$

Whereas

$$C_{Barrier}^{E,TargetX} = C_{Barrier}^{E,comp} = 0 \tag{96}$$

Transit compartments in brain meningeal cells:

$$\frac{dP1_Z}{dt} = K_{prod}^{Brain} \cdot C_Z^{MEN,N,AAV} - \frac{1}{\tau_{Brain}} \cdot P1_Z \tag{97}$$

$$\frac{dP2_Z}{dt} = \frac{1}{\tau_{Brain}} \cdot P1_Z - \frac{1}{\tau_{Brain}} \cdot P2_Z \tag{98}$$

$$\frac{dP3_Z}{dt} = \frac{1}{\tau_{Brain}} \cdot P2_Z - \frac{1}{\tau_{Brain}} \cdot P3_Z \quad (99)$$

Produced intracellular mAb concentrations in meningeal cells:

$$\frac{dC_Z^{MEN,mAb}}{dt} = \frac{1}{\tau_{org}} \cdot P3_Z - K_{sec}^{Brain} \cdot C_Z^{MEN,mAb} \quad (100)$$

Whereas

$$C_Z^{MEN,TargetX} = C_Z^{MEN,comp} = 0 \quad (101)$$

In the following section, we describe disposition of anti-TargetX mAb ("mAb"), TargetX ("$TargetX$"), and the mAb-target complex ("$comp$"), which are generalized as "SUB"for simplicity of the demonstration of equations.

"SUB" in vascular space of organs other than brain:

$$V_{org}^{V} \frac{dC_{org}^{V,SUB}}{dt} = Mass_{org}^{VI,SUB} - Mass_{org}^{VO,SUB} - \left(1 - \sigma_{org}^{V,SUB}\right) \cdot J_{org} \cdot C_{org}^{V,SUB} - CL_{up}^{org} \cdot C_{org}^{V,SUB} + CL_{up}^{org} \cdot FR \cdot C_{org}^{E,B,SUB} - Diff_{SUB} \cdot J_{org} \cdot (C_{org}^{V,SUB} - C_{org}^{I,SUB}) + 0.5 \times K_{sec}^{org} \cdot C_{org}^{E,SUB} \cdot V_{org}^{CV,E} + Binding_{org}^{V,SUB} \cdot V_{org}^{V} \quad (102)$$

Where for lung,

$$Mass_{Lung}^{VI,SUB} = (Q_{Lung} + J_{Lung}) \cdot C_{Plasma}^{SUB} \quad (103)$$

$$Mass_{Lung}^{VO,SUB} = Q_{Lung} \cdot C_{Lung}^{V,SUB} \quad (104)$$

For liver,

$$Mass_{Liver}^{VI,SUB} = Q_{Liver} \cdot C_{Lung}^{V,SUB} + \sum_{i=Pancreas,SI,LI,spleen} (Q_i - J_i) \cdot C_i^{V,SUB} \quad (105)$$

$$Mass_{Liver}^{VO,SUB} = ((Q_{Liver} - J_{Liver}) + \sum_{i=Pancreas,SI,LI,spleen} (Q_i - J_i)) \cdot C_{Liver}^{V,SUB} \quad (106)$$

For kidney,

$$Mass_{Kidney}^{VI,SUB} = Q_{Kidney} \cdot C_{Lung}^{V,SUB} \quad (107)$$

$$Mass_{Kidney}^{VO,SUB} = \left(Q_{Kidney} - J_{Kidney} + CL_R^{SUB}\right) \cdot C_{Kidney}^{V,SUB} \quad (108)$$

For all other organs,

$$Mass_{org}^{VI,SUB} = Q_{org} \cdot C_{Lung}^{V,SUB} \quad (109)$$

$$Mass_{org}^{VO,SUB} = \left(Q_{org} - J_{org}\right) \cdot C_{org}^{V,SUB} \quad (110)$$

Binding between anti-TargetX mAb and TargetX occurs in all liquid compartments:

$$Binding_{org}^{X,mAb} = -K_{on}^{mAb,TargetX} \cdot C_{org}^{X,mAb} \cdot C_{org}^{X,TargetX} + K_{off}^{mAb,TargetX} \cdot C_{org}^{X,comp} \quad (111)$$

$$Binding_{org}^{X,TargetX} = -K_{on}^{mAb,TargetX} \cdot C_{org}^{X,mAb} \cdot C_{org}^{X,TargetX} + K_{off}^{mAb,TargetX} \cdot C_{org}^{X,comp} \quad (112)$$

$$Binding_{org}^{X,comp} = K_{on}^{mAb,TargetX} \cdot C_{org}^{X,mAb} \cdot C_{org}^{X,TargetX} - K_{off}^{mAb,TargetX} \cdot C_{org}^{X,comp} \quad (113)$$

Where "X" can represent "V" and "I" in all organs and "LV", "TFV", "CM", "SAS", "PV, Art", "PV, Ven" in brain.

Interstitial "SUB" in organs other than brain:

$$V_{org}^{I}\frac{dC_{org}^{I,SUB}}{dt}=\left(1-\sigma_{org}^{V,SUB}\right)\cdot J_{org}\cdot C_{org}^{V,SUB}-\left(1-\sigma_{org}^{I}\right)\cdot J_{org}\cdot C_{org}^{I,SUB}-CL_{up}^{org}\cdot C_{org}^{I,SUB}$$
$$+CL_{up}^{org}\cdot(1-FR)\cdot C_{org}^{E,B,SUB}+Diff_{SUB}\cdot J_{org}\cdot\left(C_{org}^{V,SUB}-C_{org}^{I,SUB}\right)+K_{sec}^{org}$$
$$\cdot C_{org}^{P,SUB}\cdot V_{org}^{CV,P}+0.5\times K_{sec}^{org}\cdot C_{org}^{E,SUB}\cdot V_{org}^{CV,E}+Binding_{org}^{I,SUB}\cdot V_{org}^{I} \tag{114}$$

FcRn-unbound endosomal "SUB" in organs other than brain:

$$V_{org}^{E}\frac{dC_{org}^{E,UB,SUB}}{dt}=CL_{up}^{org}\cdot\left(C_{org}^{I,SUB}+C_{org}^{V,SUB}\right)-K_{on}^{FcRn,SUB}\cdot C_{org}^{E,UB,SUB}\cdot FcRn_{org}\cdot V_{org}^{E}$$
$$+K_{off}^{FcRn,SUB}\cdot C_{org}^{E,B,SUB}\cdot V_{org}^{E}-K_{deg}\cdot C_{org}^{E,UB,SUB}\cdot V_{org}^{E} \tag{115}$$

Endosomal FcRn-bound "SUB" concentration in organs other than brain:

$$V_{org}^{E}\frac{dC_{org}^{E,B,SUB}}{dt}=-CL_{up}^{org}\cdot C_{org}^{E,B,SUB}+K_{on}^{FcRn,SUB}\cdot C_{org}^{E,UB,SUB}\cdot FcRn_{org}\cdot V_{org}^{E}-K_{off}^{FcRn,SUB}$$
$$\cdot C_{org}^{E,B,SUB}\cdot V_{org}^{E} \tag{116}$$

Endosomal free FcRn concentration in organs other than brain:

$$V_{org}^{E}\frac{dFcRn_{org}}{dt}=\sum_{SUB=mAb,\alpha syn,comp}(CL_{up}^{org}\cdot C_{org}^{E,B,SUB}+K_{off}^{FcRn,SUB}\cdot C_{org}^{E,B,SUB}\cdot V_{org}^{E}$$
$$-K_{on}^{FcRn,SUB}\cdot C_{org}^{E,UB,SUB}\cdot FcRn_{org}\cdot V_{org}^{E}) \tag{117}$$

$$FcRn_{org}(0)=FcRn\_0$$

Interstitial "SUB" in brain:

$$V_{Brain}^{I}\frac{dC_{Brain}^{I,SUB}}{dt} = \left(1-\sigma_{BBB}^{V,SUB}\right)\cdot Q_{ISF}\cdot C_{Brain}^{V,SUB} - \left(1-\sigma_{Brain}^{I}\right)\cdot Q_{ISF}\cdot C_{Brain}^{I,SUB}$$
$$- \left(CL_{up,brain}\cdot V_{Brain}^{E}\cdot\frac{SA_{BBB}}{SA_{BBB}+SA_{BCSFB}}\right)\cdot C_{Brain}^{I,SUB}$$
$$+ \left(CL_{up,brain}\cdot V_{Brain}^{E}\cdot\frac{SA_{BBB}}{SA_{BBB}+SA_{BCSFB}}\right)\cdot(1-FR)\cdot C_{BBB}^{E,B,SUB} - CL_{up,brain,cell} \quad (118)$$
$$\cdot C_{Brain}^{I,SUB} + PS_{PV}^{SUB}\cdot\left(C_{Brain}^{PV,Art,SUB} - C_{Brain}^{I,SUB}\right) + PS_{PV}^{SUB}\cdot\left(C_{Brain}^{PV,Ven,SUB} - C_{Brain}^{I,SUB}\right)$$
$$+ Binding_{Brain}^{I,SUB}\cdot V_{Brain}^{I} + Ksyn_{I}^{SUB}\cdot V_{Brain}^{I} + K_{sec}^{Brain}\cdot C_{Brain}^{P,SUB}\cdot V_{Brain}^{CV,P}$$
$$+ 0.5\times K_{sec}^{Brain}\cdot C_{BBB}^{E,SUB}\cdot V_{Brain}^{CV,E}\cdot\frac{SA_{BBB}}{SA_{BBB}+SA_{BCSFB}}$$

Vascular "SUB" in brain:

$$V_{Brain}^{V}\frac{dC_{Brain}^{V,SUB}}{dt} = Q_{Brain}\cdot C_{Lung}^{V,SUB} - (Q_{Brain}-Q_{ISF}-Q_{CSF})\cdot C_{Brain}^{V,SUB} - \left(1-\sigma_{BBB}^{V,SUB}\right)\cdot Q_{ISF}$$
$$\cdot C_{Brain}^{V,SUB} - \left(1-\sigma_{BCSFB}^{V,SUB}\right)\cdot Q_{CSF}\cdot C_{Brain}^{V,SUB} - \left(CL_{up,brain}\cdot V_{Brain}^{E}\right)\cdot C_{Brain}^{V,SUB}$$
$$+ \left(CL_{up,brain}\cdot V_{Brain}^{E}\cdot\frac{SA_{BBB}}{SA_{BBB}+SA_{BCSFB}}\right)\cdot FR\cdot C_{BBB}^{E,B,SUB}$$
$$+ \left(CL_{up,brain}\cdot V_{Brain}^{E}\cdot\frac{SA_{BCSFB}}{SA_{BBB}+SA_{BCSFB}}\right)\cdot FR\cdot C_{BCSFB}^{E,B,SUB} + 0.5\times K_{sec}^{Brain} \quad (119)$$
$$\cdot C_{BBB}^{E,SUB}\cdot V_{Brain}^{CV,E}\cdot\frac{SA_{BBB}}{SA_{BBB}+SA_{BCSFB}} + 0.5\times K_{sec}^{Brain}\cdot C_{BCSFB}^{E,SUB}\cdot V_{Brain}^{CV,E}$$
$$\cdot\frac{SA_{BCSFB}}{SA_{BBB}+SA_{BCSFB}} + Binding_{Brain}^{V,SUB}\cdot V_{Brain}^{V}$$

FcRn-unbound "SUB" in endosomal space at BBB:

$$V_{Brain}^{BBB}\frac{dC_{BBB}^{E,UB,SUB}}{dt}$$

$$= (CL_{up,brain}\cdot V_{Brain}^{E}\cdot\frac{SA_{BBB}}{SA_{BBB}+SA_{BCSFB}})\cdot\left(C_{Brain}^{V,SUB}+C_{Brain}^{I,SUB}\right)-K_{on}^{FcRn,SUB}$$
$$\cdot C_{BBB}^{E,UB,SUB}\cdot FcRn_{BBB}\cdot V_{Brain}^{BBB}+K_{off}^{FcRn,SUB}\cdot C_{BBB}^{E,B,SUB}\cdot V_{Brain}^{BBB}-K_{deg}$$
$$\cdot C_{BBB}^{E,UB,SUB}\cdot V_{Brain}^{BBB} \quad (120)$$

FcRn bound "SUB" in endosomal space at BBB:

$$V_{Brain}^{BBB}\frac{dC_{BBB}^{E,B,SUB}}{dt}$$

$$= -(CL_{up,brain}\cdot V_{Brain}^{E}\cdot\frac{SA_{BBB}}{SA_{BBB}+SA_{BCSFB}})\cdot C_{BBB}^{E,B,SUB}+K_{on}^{FcRn,SUB} \quad (121)$$
$$\cdot C_{BBB}^{E,UB,SUB}\cdot FcRn_{BBB}\cdot V_{Brain}^{BBB}-K_{off}^{FcRn,SUB}\cdot C_{BBB}^{E,B,SUB}\cdot V_{Brain}^{BBB}$$

Unbound FcRn concentration in BBB:

$$V_{Brain}^{BBB}\frac{dFcRn_{BBB}}{dt}$$

$$= \sum_{SUB=mAb,TargetX,comp}((CL_{up,brain}\cdot V_{Brain}^{E}\cdot\frac{SA_{BBB}}{SA_{BBB}+SA_{BCSFB}})\cdot C_{BBB}^{E,B,SUB} \quad (122)$$
$$-K_{on}^{FcRn,SUB}\cdot C_{BBB}^{E,UB,SUB}\cdot FcRn_{BBB}\cdot V_{Brain}^{BBB}+K_{off}^{FcRn,SUB}\cdot C_{BBB}^{E,B,SUB}\cdot V_{Brain}^{BBB})$$

$$FcRn_{BBB}(0) = FcRn\_0$$

FcRn-unbound "SUB" in endosomal space at BCSFB:

$$
V_{Brain}^{BCSFB} \frac{dC_{BCSFB}^{E,UB,SUB}}{dt}
$$

$$
\begin{aligned}
&= (CL_{up,brain} \cdot V_{Brain}^{E} \cdot \frac{SA_{BCSFB}}{SA_{BBB} + SA_{BCSFB}}) \cdot C_{Brain}^{V,SUB} + (CL_{up,brain} \cdot V_{Brain}^{E} \\
&\cdot \frac{SA_{BCSFB}}{SA_{BBB} + SA_{BCSFB}} \cdot \frac{V_{Brain}^{LV}}{V_{Brain}^{LV} + V_{Brain}^{TFV}}) \cdot C_{Brain}^{LV,SUB} + (CL_{up,brain} \cdot V_{Brain}^{E} \\
&\cdot \frac{SA_{BCSFB}}{SA_{BBB} + SA_{BCSFB}} \cdot \frac{V_{Brain}^{TFV}}{V_{Brain}^{LV} + V_{Brain}^{TFV}}) \cdot C_{Brain}^{TFV,SUB} - K_{on}^{FcRn,SUB} \cdot C_{BCSFB}^{E,UB,SUB} \\
&\cdot FcRn_{BCSFB} \cdot V_{Brain}^{BCSFB} + K_{off}^{FcRn,SUB} \cdot C_{BCSFB}^{E,B,SUB} \cdot V_{Brain}^{BCSFB} - K_{deg} \cdot C_{BCSFB}^{E,UB,SUB} \\
&\cdot V_{Brain}^{BCSFB}
\end{aligned} \tag{123}
$$

FcRn bound "SUB" in endosomal space at BCSFB:

$$
V_{Brain}^{BCSFB} \frac{dC_{BCSFB}^{E,B,SUB}}{dt}
$$

$$
\begin{aligned}
&= K_{on}^{FcRn,SUB} \cdot C_{BCSFB}^{E,UB,SUB} \cdot FcRn_{BCSFB} \cdot V_{Brain}^{BCSFB} - K_{off}^{FcRn,SUB} \cdot C_{BCSFB}^{E,B,SUB} \cdot V_{Brain}^{BCSFB} \\
&- (CL_{up,brain} \cdot V_{Brain}^{E} \cdot \frac{SA_{BCSFB}}{SA_{BBB} + SA_{BCSFB}}) \cdot C_{BCSFB}^{E,B,SUB}
\end{aligned} \tag{124}
$$

Free FcRn at BCSFB:

$$
V_{Brain}^{BCSFB} \frac{dFcRn_{BCSFB}}{dt}
$$

$$
\begin{aligned}
&= \sum_{SUB=mAb,TargetX,comp} ((CL_{up,brain} \cdot V_{Brain}^{E} \cdot \frac{SA_{BCSFB}}{SA_{BBB} + SA_{BCSFB}}) \cdot C_{BCSFB}^{E,B,SUB} \\
&- K_{on}^{FcRn,SUB} \cdot C_{BCSFB}^{E,UB,SUB} \cdot FcRn_{BCSFB} \cdot V_{Brain}^{BCSFB} + K_{off}^{FcRn,SUB} \cdot C_{BCSFB}^{E,B,SUB} \\
&\cdot V_{Brain}^{BCSFB})
\end{aligned} \tag{125}
$$

$$
FcRn_{BCSFB}(0) = FcRn\_0
$$

"SUB" in LV:

$$V_{Brain}^{LV}\frac{dC_{Brain}^{LV,SUB}}{dt} = \left(1-\sigma_{BCSFB}^{V,SUB}\right)\cdot\left(Q_{CSF}\cdot\frac{V_{Brain}^{LV}}{V_{Brain}^{LV}+V_{Brain}^{TFV}}\right)\cdot C_{Brain}^{V,SUB}-\left(1-\sigma_{CSF}^{LV-TFV,SUB}\right)\cdot\left(Q_{CSF}\cdot\frac{V_{Brain}^{LV}}{V_{Brain}^{LV}+V_{Brain}^{TFV}}\right)\cdot C_{Brain}^{LV,SUB}-\left(CL_{up,brain}\cdot V_{Brain}^{E}\cdot\frac{SA_{BCSFB}}{SA_{BBB}+SA_{BCSFB}}\cdot\frac{V_{Brain}^{LV}}{V_{Brain}^{LV}+V_{Brain}^{TFV}}\right)\cdot C_{Brain}^{LV,SUB}+\left(CL_{up,brain}\cdot V_{Brain}^{E}\cdot\frac{SA_{BCSFB}}{SA_{BBB}+SA_{BCSFB}}\cdot\frac{V_{Brain}^{LV}}{V_{Brain}^{LV}+V_{Brain}^{TFV}}\right)\cdot(1-FR)\cdot C_{BCSFB}^{E,B,SUB}+Binding_{Brain}^{LV,SUB}\cdot V_{Brain}^{LV}+0.5\times K_{sec}^{Brain}\cdot C_{BCSFB}^{E,SUB}\cdot V_{Brain}^{CV,E}\cdot\frac{SA_{BCSFB}}{SA_{BBB}+SA_{BCSFB}}\cdot\frac{V_{Brain}^{LV}}{V_{Brain}^{LV}+V_{Brain}^{TFV}}+Ksyn_{LV}^{SUB}\cdot V_{Brain}^{LV} \tag{126}$$

"SUB" in TFV:

$$V_{Brain}^{TFV}\frac{dC_{Brain}^{TFV,SUB}}{dt} = \left(1-\sigma_{BCSFB}^{V,SUB}\right)\cdot\left(Q_{CSF}\cdot\frac{V_{Brain}^{TFV}}{V_{Brain}^{LV}+V_{Brain}^{TFV}}\right)\cdot C_{Brain}^{V,SUB}+\left(1-\sigma_{CSF}^{LV-TFV,SUB}\right)\cdot\left(Q_{CSF}\cdot\frac{V_{Brain}^{LV}}{V_{Brain}^{LV}+V_{Brain}^{TFV}}\right)\cdot C_{Brain}^{LV,SUB}-\left(1-\sigma_{CSF}^{TFV-CM,SUB}\right)\cdot Q_{CSF}\cdot C_{Brain}^{TFV,SUB}-\left(CL_{up,brain}\cdot V_{Brain}^{E}\cdot\frac{SA_{BCSFB}}{SA_{BBB}+SA_{BCSFB}}\cdot\frac{V_{Brain}^{TFV}}{V_{Brain}^{LV}+V_{Brain}^{TFV}}\right)\cdot C_{Brain}^{TFV,SUB}+\left(CL_{up,brain}\cdot V_{Brain}^{E}\cdot\frac{SA_{BCSFB}}{SA_{BBB}+SA_{BCSFB}}\cdot\frac{V_{Brain}^{TFV}}{V_{Brain}^{LV}+V_{Brain}^{TFV}}\right)\cdot(1-FR)\cdot C_{BCSFB}^{E,B,SUB}+Binding_{Brain}^{TFV,SUB}\cdot V_{Brain}^{TFV}+0.5\times K_{sec}^{Brain}\cdot C_{BCSFB}^{E,SUB}\cdot V_{Brain}^{CV,E}\cdot\frac{SA_{BCSFB}}{SA_{BBB}+SA_{BCSFB}}\cdot\frac{V_{Brain}^{TFV}}{V_{Brain}^{LV}+V_{Brain}^{TFV}} \tag{127}$$

"SUB" in CM:

$$V_{Brain}^{CM}\frac{dC_{Brain}^{CM,SUB}}{dt} = \left(1-\sigma_{CSF}^{TFV-CM,SUB}\right)\cdot Q_{CSF}\cdot C_{Brain}^{TFV,SUB} + Q_{CSF}^{OSC}\cdot C_{Brain}^{SAS,SUB} - \left(1-\sigma_{CSF}^{CM-SAS,SUB}\right)\cdot\left(Q_{CSF}+Q_{CSF}^{OSC}\right)\cdot C_{Brain}^{CM,SUB} + Binding_{Brain}^{CM,SUB}\cdot V_{Brain}^{CM} + K_{sec}^{Brain}\cdot C_{CM}^{MEN,mAb}\cdot\left(V_{Brain}^{MEN}\cdot\frac{V_{Brain}^{CM}}{V_{Brain}^{CM}+V_{Brain}^{SAS}}\right) \tag{128}$$

"SUB" in SAS:

$$V_{Brain}^{SAS}\frac{dC_{Brain}^{SAS,SUB}}{dt} = \left(1-\sigma_{CSF}^{CM-SAS,SUB}\right)\cdot\left(Q_{CSF}+Q_{CSF}^{OSC}\right)\cdot C_{Brain}^{CM,SUB} - (1-\sigma_{SAS}^{I})\cdot Q_{CSF}\cdot C_{Brain}^{SAS,SUB} - Q_{CSF}^{OSC}\cdot C_{Brain}^{SAS,SUB} - Q_{PV}\cdot C_{Brain}^{SAS,SUB} + Q_{PV}\cdot C_{Brain}^{PV,Ven,SUB} + Binding_{Brain}^{SAS,SUB}\cdot V_{Brain}^{SAS} + K_{sec}^{Brain}\cdot C_{SAS}^{MEN,mAb}\cdot(V_{Brain}^{MEN}\cdot\frac{V_{Brain}^{SAS}}{V_{Brain}^{CM}+V_{Brain}^{SAS}}) \tag{129}$$

"SUB" in perivascular arterial space:

$$V_{Brain}^{PV,Art}\frac{dC_{Brain}^{PV,Art,SUB}}{dt} = Q_{PV}\cdot C_{Brain}^{SAS,SUB} - PS_{PV}^{SUB}\cdot\left(C_{Brain}^{PV,Art,SUB}-C_{Brain}^{I,SUB}\right) + Binding_{Brain}^{PV,Art,SUB}\cdot V_{Brain}^{PV,Art} \tag{130}$$

$$V_{Brain}^{PV,Ven}\frac{dC_{Brain}^{PV,Ven,SUB}}{dt} = \left(1-\sigma_{Brain}^{I}\right)\cdot Q_{ISF}\cdot C_{Brain}^{I,SUB} - (Q_{PV}+Q_{ISF})\cdot C_{Brain}^{PV,Ven,SUB} - PS_{PV}^{SUB}\cdot\left(C_{Brain}^{PV,Ven,SUB}-C_{Brain}^{I,SUB}\right) + Binding_{Brain}^{PV,Ven,SUB}\cdot V_{Brain}^{PV,Ven} \tag{131}$$

Endosomal "SUB" in brain parenchymal cells via nonspecific pinocytosis:

$$V_{Brain}^{P,Endo}\frac{dC_{Brain}^{P,E,SUB}}{dt} = CL_{up,brain,cell}\cdot C_{Brain}^{I,SUB} - K_{deg}\cdot C_{Brain}^{P,E,SUB}\cdot V_{Brain}^{P,Endo} \tag{132}$$

“SUB” in central plasma:

$$\begin{aligned} V_{Plasma}\frac{dC_{Plasma}^{SUB}}{dt} \\ &= \sum_{\substack{i=All\ organs\ excluding\ brain,lung,\\ liver,SI,LI,pancreas,spleen}} (Q_i - J_i)\cdot C_i^{V,SUB} \\ &+ (Q_{Brain} - Q_{ISF} - Q_{CSF})\cdot C_{Brain}^{V,SUB} \\ &+ \left((Q_{Liver} - J_{Liver}) + \sum_{i=Pancreas,SI,LI,spleen}(Q_i - J_i)\right)\cdot C_{Liver}^{V,SUB} + L_{Lymph} \\ &\cdot C_{Lymph}^{SUB} - \left(Q_{Lung} + J_{Lung}\right)\cdot C_{Plasma}^{SUB} + Binding_{Plasma}^{SUB}\cdot V_{Plasma} \\ &+ Ksyn_{Plasma}^{SUB}\cdot V_{Plasma} \end{aligned} \tag{133}$$

In particular,

$$Binding_Y^{mAb} = -K_{on}^{mAb,TargetX}\cdot C_{Plasma}^{mAb}\cdot C_{Plasma}^{TargetX} + K_{off}^{mAb,TargetX}\cdot C_{Plasma}^{comp} \tag{134}$$

$$Binding_Y^{TargetX} = -K_{on}^{mAb,TargetX}\cdot C_{Plasma}^{mAb}\cdot C_{Plasma}^{TargetX} + K_{off}^{mAb,TargetX}\cdot C_{Plasma}^{comp} \tag{135}$$

$$Binding_Y^{comp} = K_{on}^{mAb,TargetX}\cdot C_{Plasma}^{mAb}\cdot C_{Plasma}^{TargetX} - K_{off}^{mAb,TargetX}\cdot C_{Plasma}^{comp} \tag{136}$$

Where "Y" is "$Plasma$" or "$Lymph$".

“SUB” in the lymph:

$$V_{Lymph}\frac{dC_{Lymph}^{SUB}}{dt} = \sum_{i=All\ organs\ excluding\ brain}\left(1-\sigma_i^I\right)\cdot J_i\cdot C_i^{I,SUB}+Q_{ISF}\cdot C_{Brain}^{PV,Ven,SUB} + \left(1-\sigma_{SAS}^I\right)\cdot Q_{CSF}\cdot C_{Brain}^{SAS,SUB}-L_{Lymph}\cdot C_{Lymph}^{SUB}+Binding_{Lymph}^{SUB}\cdot V_{Lymph} \quad (137)$$

References

1. Wu, S., et al., *Investigation of Antibody Pharmacokinetics in the Brain Following Intra-CNS Administration and Development of PBPK Model to Characterize the Data.* AAPS J, 2024. **26**(2): p. 29.
2. Li, Z., et al., *Effect of Size on Solid Tumor Disposition of Protein Therapeutics.* Drug Metab Dispos, 2019. **47**(10): p. 1136-1145.
3. Sender, R., S. Fuchs, and R. Milo, *Revised Estimates for the Number of Human and Bacteria Cells in the Body.* PLoS Biol, 2016. **14**(8): p. e1002533.
4. Pearson, W.R., J.R. Wu, and J. Bonner, *Analysis of rat repetitive DNA sequences.* Biochemistry, 1978. **17**(1): p. 51-9.
